# The human genome contains over a million autonomous exons

**DOI:** 10.1101/gr.277792.123

**Published:** 2023-11

**Authors:** Nicholas Stepankiw, Ally W.H. Yang, Timothy R. Hughes

**Affiliations:** 1 Donnelly Centre, University of Toronto, Toronto, Ontario, Canada M5S 3E1;; 2 Department of Molecular Genetics, University of Toronto, Toronto, Ontario, Canada M5S 1A8

## Abstract

Mammalian mRNA and lncRNA exons are often small compared to introns. The exon definition model predicts that exons splice autonomously, dependent on proximal exon sequence features, explaining their delineation within large introns. This model has not been examined on a genome-wide scale, however, leaving open the question of how often mRNA and lncRNA exons are autonomous. It is also unknown how frequently such exons can arise by chance. Here, we directly assayed large fragments (500–1000 bp) of the human genome by exon trapping, which detects exons spliced into a heterologous transgene, here designed with a large intron context. We define the trapped exons as “autonomous.” We obtained ∼1.25 million trapped exons, including most known mRNA and well-annotated lncRNA internal exons, demonstrating that human exons are predominantly autonomous. mRNA exons are trapped with the highest efficiency. Nearly a million of the trapped exons are unannotated, most located in intergenic regions and antisense to mRNA, with depletion from the forward strand of introns. These exons are not conserved, suggesting they are nonfunctional and arose from random mutations. They are nonetheless highly enriched with known splicing promoting sequence features that delineate known exons. Novel autonomous exons are more numerous than annotated lncRNA exons, and computational models also indicate they will occur with similar frequency in any randomly generated sequence. These results show that most human coding exons splice autonomously, and provide an explanation for the existence of many unconserved lncRNAs, as well as a new annotation and inclusion levels of spliceable loci in the human genome.

Eukaryotic primary transcripts, including those of most human coding genes, are often composed of alternating exons and introns. In human, and most vertebrates, the introns are generally much larger than the exons (International Human Genome Sequencing Consortium 2001). The intron/exon boundaries consist of relatively short and degenerate 5′ and 3′ splice site sequences (hereafter, 5′ and 3′SS, respectively), and due to random chance, large introns will contain many sequences that resemble 5′ and 3′SS (Sun and Chasin 2000; Côté et al. 2001; Yeo and Burge 2004). Precise removal of introns is thought to be facilitated mainly by a mechanism known as exon definition (Robberson et al. 1990; Piovesan et al. 2019), in which the recognition of adjacent flanking 3′ and 5′SS are facilitated by bridging of the splicing complexes across the exon. The specificity gained by the characteristic 80–220 base spacing between the 3′ and 5′SS is insufficient to precisely specify human exons, however, as many exons are outside this range. Presumably as a consequence, human exons are often associated with additional sequences that promote inclusion (known as splicing enhancers) (Liu et al. 1998; Reed 2000; Côté et al. 2001; Wang et al. 2004b; Zhang et al. 2005; Ke et al. 2011; Wang et al. 2012). Exon splicing therefore depends on a variety of sequence features, with the 3′SS and 5′SS being essential. The sequence features that delineate exons remain incompletely known, however, and as a result, the exon definition model has not been explicitly confirmed on a genomic scale. Thus, it remains unknown what proportion of human exons are autonomous, that is, containing sequences that are sufficient to enable splicing into a mature transcript.

A variety of computational approaches have been taken to predict exon identity and inclusion level from primary sequence. These algorithms would presumably learn or incorporate features that are used by cells to delineate exons, but they also tend to include additional correlated information that is not relevant to mechanistic understanding of exon recognition. Thus, they do not explicitly predict exon autonomy, nor do they reveal the required sequence features. For example, gene-finding programs perform this task (e.g., GenScan [Burge and Karlin 1997]), but these typically incorporate coding potential, sequence conservation, and other factors (see Scalzitti et al. 2020 for a recent overview). Much of the literature has focused on predicting inclusion levels of alternative exons (Cartegni et al. 2003; Fairbrother et al. 2004; Barash et al. 2010; Rosenberg et al. 2015; Xiong et al. 2015) but these methods assume exon boundaries are known. Most coding exons are constitutive, in any case (Pan et al. 2008; Wang et al. 2008); it is not clear that alternative splicing signals would be the same signals that define the exons. SpliceAI, a well-known predictor of splice sites, operates directly from primary sequence, using Convolutional Neural Networks (CNNs) trained on splice site locations within known mRNA genes, thus presumably incorporating local context (Jaganathan et al. 2019). SpliceAI captures splice sites of both constitutive and alternative exons, but it does not directly report the identity of full exons. In addition, as a CNN with ∼700,000 parameters, it is inherently challenging to interpret. Moreover, exon-associated sequence features (e.g., those that would contribute to protein-coding ability or transcript stability, and not splicing per se) may contribute to computational discrimination of annotated exons versus other sequences, without necessarily being mechanistic drivers of splicing itself.

A related fundamental question is how many exons (broadly defined as sequences that can splice into a mature transcript) exist in the human genome. There are ∼181,000 annotated internal exons within the ∼20,000 known human protein-coding genes; these constitute roughly 1% of the human genome. An even larger fraction may comprise the enigmatic lncRNAs (long noncoding RNAs), however. In aggregate, upwards of 800,000 lncRNA exons have been cataloged, of which at least 250,000 are internal exons (Lagarde et al. 2017; The RNAcentral Consortium et al. 2017; Pertea et al. 2018; Volders et al. 2019; Zhao et al. 2021). In contrast, only ∼25,000 internal exons are annotated as part of a lncRNA in the curated ENCODE collection (GENCODE v37 [Frankish et al. 2019]). Many lncRNAs appear to be extremely rare, as they are found at low levels, or in only one data set. The vast majority of lncRNAs have no known function (Ponting and Haerty 2022), and many display weaker splicing signals than protein-coding genes (Deveson et al. 2018). It has been proposed that many arise as transcriptional “noise” (Ponting and Haerty 2022), which could arise as a consequence of transcription from enhancers (Engreitz et al. 2016). While eRNAs (enhancer RNAs) are typically unstable, well-annotated lncRNAs typically contain multiple exons (Orom et al. 2010), presumably stabilizing the lncRNAs, as splicing signals are known to enhance both transcription and RNA stability (Le Hir et al. 2003; Core et al. 2014).

The classical exon definition model predicts that exons would be largely self-determined by local sequence features, but the fact that splicing does not always occur in a strictly linear fashion along primary transcripts (e.g., Drexler et al. 2020) suggests the possibility of distant interactions and dependency among exons and flanking genomic features. We therefore sought to survey the human genome to ask which exons are “autonomous” (i.e., self-defined, as they will splice into a heterologous transgene with relatively large introns). We used a classical “exon trapping” assay to survey the human genome for autonomous exons (Duyk et al. 1990) whereby genomic fragments are assayed outside of their normal contextual setting, for example, flanking exons, promoter, transcription level, and distal intronic sequences. We reasoned that this survey would allow us to query whether protein-coding exons are generally autonomous, whether exons exist elsewhere in the genome, what sequence features they possess, and whether exons arise at random, which would partly explain the existence of lncRNAs. The results clarify several aspects of the human exon complement, identify a large number of previously undocumented exons, and indicate that multiexon lncRNAs are an expected feature of large genomes.

## Results

### Genome-wide exon trapping

**Figure 1.**
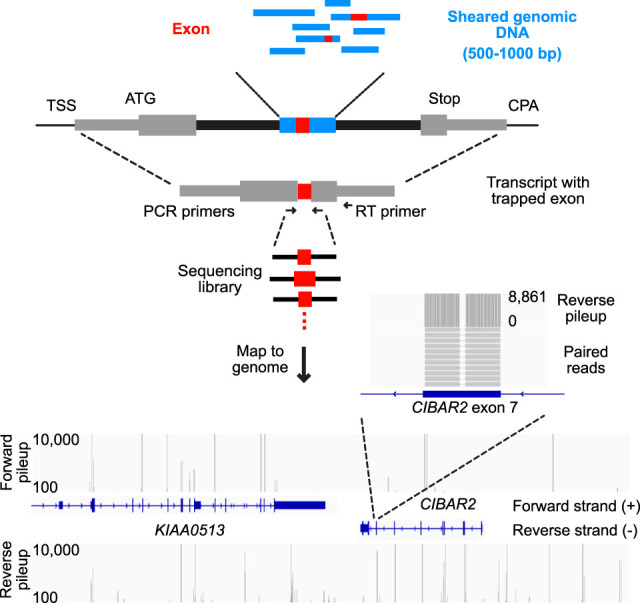
Overview of the genome exon trapping method. Diagram depicting exon trapping approach, sequencing library construction and example sequencing read maps. Sheared genomic DNA library fragments (blue boxes) 500–1000 bp in length are cloned into the middle of the sixth intron from *TRA2B* (black boxes), in a pcDNA 3.1 vector backbone. First and terminal exons (gray boxes) are labeled with the transcriptional start site (TSS), start codon (ATG), stop codon (Stop), and the cleavage and polyadenylation site (CPA). Internal exons (red boxes) are amplified by RT-PCR, using indicated primers, then sequenced and mapped to the human genome (hg38). *Bottom* panel shows mapped sequencing read counts (separated into forward and reverse strand pileups) for regions containing KIAA0513 and a portion of *CIBAR2* (display region coordinates: Chr 16: 85,062,938–85,134,585). The zoom-in region corresponds to exon 7 of *CIBAR2*.

We used a classical exon trapping assay, in which a query sequence is cloned into the middle of a 1.6 kb intron, to survey the human genome (see Fig. 1 for a schematic overview and example data). If the query sequence contains an autonomous exon, that exon (or more than one exon) will be included in the resulting spliced transcript. We used five vectors that differ in either reading frame relative to the splice sites (0,+1,+2), removal of a predicted downstream intronic splicing enhancer sequence (a binding site for RBFOX1 within a hairpin), or a 5′SS mutation that we engineered to weaken the predicted splice site strength (see Methods, Supplemental Fig. S1A). These variations in the splicing context were included initially to ask whether gross systematic differences would result, but even those specifically sought (i.e., impact of reading frame) appeared relatively minor. We therefore pooled all of the reads, to increase numbers and provide redundancy.

Query sequences consisted of 23 libraries (four or five libraries for each vector) which were generated from sheared human genomic DNA fragments (500–1000 bp). Each of the 23 plasmid libraries consisted of 2 to 20 million bacterial clones, and was transfected into ∼2 million HEK293 cells. The number of plasmids per transfected cell was not assessed, but 2 million unique plasmids would represent roughly a quarter of the stranded genome. We therefore anticipated up to onefold genomic coverage per library, and up to fivefold sampling of the genome over all 23 libraries. Following transient transfection of reporter construct libraries, RNA extraction, and poly(A) selection, we generated reverse transcriptase–polymerase chain reaction (RT-PCR) products containing the trapped exon. We then sequenced the resulting PCR products and mapped the reads to the genome. As with other Massively Parallel Reporter Assays, we measure the splicing frequency of exons across the genome using the sequencing read counts, which we take as a proxy for the inclusion rate of the exon.

**Figure 2.**
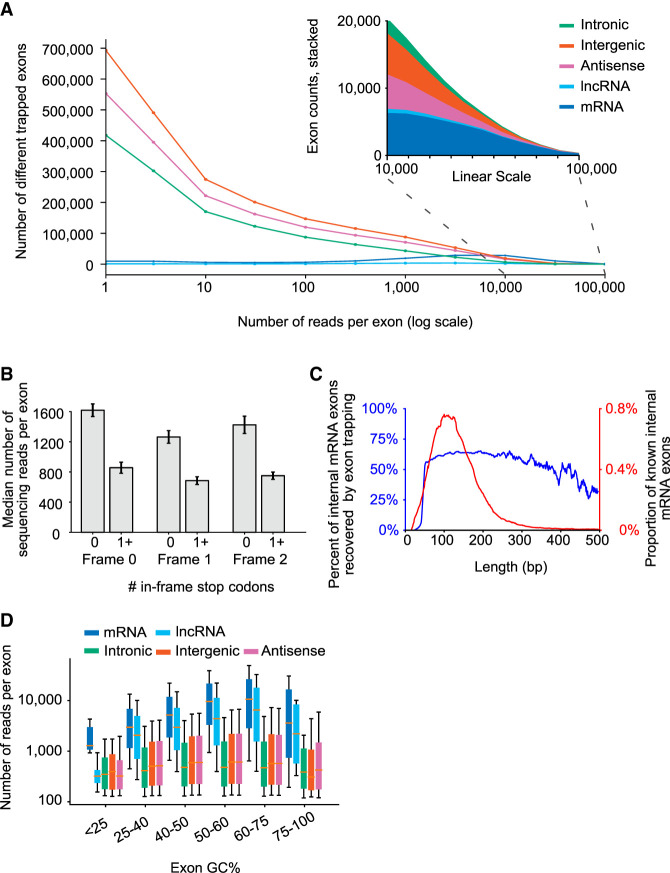
Properties of trapped exons. (*A*) Histograms of sequencing read counts for trapped internal exons within different genomic regions. *Outset* plot shows logarithmic exon counts and *inset* shows zoomed linear exon counts. Logarithmic bin boundaries indicated by dots corresponding to 10^x^ for x from 0 to 5 with step size 0.5. Linear bin boundaries range from 100 to 100,000 with a step size of 1000. (*B*) Bar plots depicting sequencing read counts of internal exons containing zero or at least one in-frame stop codon. Results for reading frames in the first (“Frame 0”), second (“Frame 1”), and third (“Frame 2”) positions are shown. (*C*) Line plots depicting the distribution of mRNA exon lengths and the percent of mRNA exons at each length recovered by exon trapping. Plots have a 9 bp smoothing window applied. (*D*) Boxplots depicting GC content of trapped internal exons from different genomic regions. *y*-axis indicates sequencing read counts of trapped exons, within indicated GC content ranges (*x*-axis). Whiskers indicate 10th and 90th percentiles.

In total, we obtained ∼4.2 billion paired end reads that mapped uniquely to the human genome (an average of ∼200 million reads per library). These reads mapped to ∼6 million clusters with identical or nearly identical ends (see Methods for details). These initial clusters encompassed ∼9% of the genome. The majority of exon clusters were supported by 10 or fewer reads, however (Fig. 2A; Supplemental Fig. S2A), and many were found in only one library (Supplemental Fig. S2B). Internal exons from protein-coding genes, in contrast, had an average of 10,636 reads, and were typically found in multiple libraries (Supplemental Fig. S2C), together encompassing 32% of all reads.

We limited most of our subsequent analyses to exon clusters with at least 100 reads across all libraries (hereafter, we refer to “exon clusters” as “exons”), with the goal of cataloging exons with very high confidence. When we randomized the positions of reads across the forward strand of a chromosome (Chr 17, chosen for its relatively small size) and identified exons using the same process described above, none exceeded 10 reads. In the original data set, however, 27,715 of the trapped exons exceeded 100 reads. Thus, 100 reads is a very conservative threshold, which we anticipate will be robust to alternative statistical tests. Across the entire genome in the real exon trapping data, a threshold of 100 reads captures 1,245,947 exons in total, encompassing 3.2% of the stranded genome (i.e., 6.2 Gb) (hereafter referred to as “1.25 million exons”). This figure is much higher than we had initially expected, and is thus the focus of this paper, beyond this section.

We retrospectively examined the detection of the 1.25 million exons in the different vectors and libraries, in order to estimate coverage. The individual vectors each captured between 41% and 75% of all sequence present in any vector (i.e., there is not even a single read for the remaining 59% and 25% of the 1.25 million exons, respectively), roughly consistent with the 60% breadth that would be expected from 1× coverage. Overlap between the vectors is consistent with random sampling (i.e., the intersection is similar to expectation if both vectors sampled from 1.25 million exons) (Supplemental Fig. S1B). The distribution of individual exons across libraries also appears random, with most of the 1.25 million exons present in multiple libraries (Supplemental Fig. S2D,E). Manual inspection of read counts on genome browser displays typically shows an “all or nothing” pattern, in which an exon is detected in a library either hundreds of times, or not at all, again consistent with incomplete sampling in individual libraries. Most of the 1.25 million exons were detected in at least half of the libraries (examples in Supplemental Fig. S2F), showing that they are not an artifact of a single library or vector. Importantly, these results do not show coverage of all possible exons in the genome; below, we describe several exon attributes that are depleted. They do, however, indicate that the vast majority of exons that would be detected in this experimental system are present in the data set. Our estimated fivefold coverage would correspond to ∼99% breadth; even threefold would correspond to over 90%.

The five different libraries did vary systematically in the inclusion of individual exons to some degree, but not as greatly as we had anticipated. The impact of nonsense mediated decay (NMD) (Maquat 2004) was clearly observed among the vectors in three different reading frames, but with only a twofold decrease, on average, associated with stop codons in the trapped exon (Fig. 2B). Read counts of identical exons, compared between two libraries—with a stop codon in one library but not the other, because of reading frame—also showed a median decrease of just over twofold (Supplemental Fig. S3A,B). Thus, NMD has a quantitative, but generally not qualitative effect in the splicing reporter assay. As our main goal in this manuscript is to provide an overall picture of the unexpectedly high number of exons observed, we pooled the reads from all libraries for subsequent analyses.

We note that the size of the genomic fragments in the libraries is large enough to accommodate two closely spaced exons, which could be captured in the assay simultaneously. If the exons are short, such cases should be detectable in single reads. Indeed, we observed 14,830 trapped exons corresponding to such mRNA “doublets,” associated with 8.2% of trapped mRNA exons. However, 9590 of these are also associated with respective single exons. Anecdotally, the same appears to be true for non-mRNA exons among the 1.25 million, but because of uncertainties in mapping from the ends of reads we did not further examine this phenomenon; extrapolating from mRNA exons, we expect that roughly 3% of new exons described here may in fact represent two adjacent exons.

### Exon inclusion rate varies among types of RNA, and with exon properties

We next determined the proportion of known exons captured (from mRNAs and lncRNAs), and where these trapped exons are found relative to known transcript structures (Lagarde et al. 2017; The RNAcentral Consortium et al. 2017; Pertea et al. 2018; Volders et al. 2019; Zhao et al. 2021). As noted above, at least a subset of known exons is very well captured: Figure 2A shows that exon clusters with very high read counts (higher than 20,000) largely correspond to internal exons from protein-coding genes, despite these exons representing less than 1% of the genome.

The cutoff of 100 reads retains the majority of GENCODE mRNA (61%) (and lncRNA [53%]) internal exons in the trapped exons. Thus, even though only a single cell line was used, the assay clearly shows that the majority of human protein-coding exons are autonomous (78% and 68% are detected at a threshold of one read for GENCODE mRNA and lncRNA, respectively).

We examined what properties of exons may control detection in exon trapping. Internal exons with high inclusion rates tend to have stronger splice sites; this expected finding is explored in more detail below. The capture rate of GENCODE mRNA internal exons also depends on exon length, and is highest between 50 and 250 bases (blue line in Fig. 2C). This range encompasses the ∼140 bp that is typical of internal exons, which presumably facilitates U2/U1 bridging (Robberson et al. 1990) (red line in Fig. 2C). The capture rate also depends strongly on GC content (Fig. 2D). We cannot rule out a technical origin for this phenomenon, but we note that base content has the potential to impact RBP binding site frequency, and could influence nucleosome occupancy (Tillo and Hughes 2009). There are many indications that nucleosomes promote splicing (Hollander et al. 2016), and indeed, exons with 50%–75% GC are captured at the highest rates (Fig. 2D). The effect of exon length is also influenced by base content, in that high GC content is associated with recovery of longer exons (Supplemental Fig. S3C,D), consistent with high GC content helping to overcome lack of U2/U1 bridging.

The internal exons of housekeeping genes (Hounkpe et al. 2021), genes expressed highly in HEK293 cells (Nieborak et al. 2023), and the exons of all other coding genes displayed similar read counts in the exon trapping data (Supplemental Fig. S4A). Exon sequencing read counts are, however, dependent on the PSI measured in HEK293 cells (Ellis et al. 2023; Supplemental Fig. S4B), and alternative HEK293 mRNA exons are frequently missing in the exon trapping data (Supplemental Fig. S4C). Alternative exons of all types have lower read counts on average (see below). Thus, altogether, sequence properties of exons themselves have a strong impact on exon trapping, but expression level of the corresponding gene has almost none.

**Figure 3.**
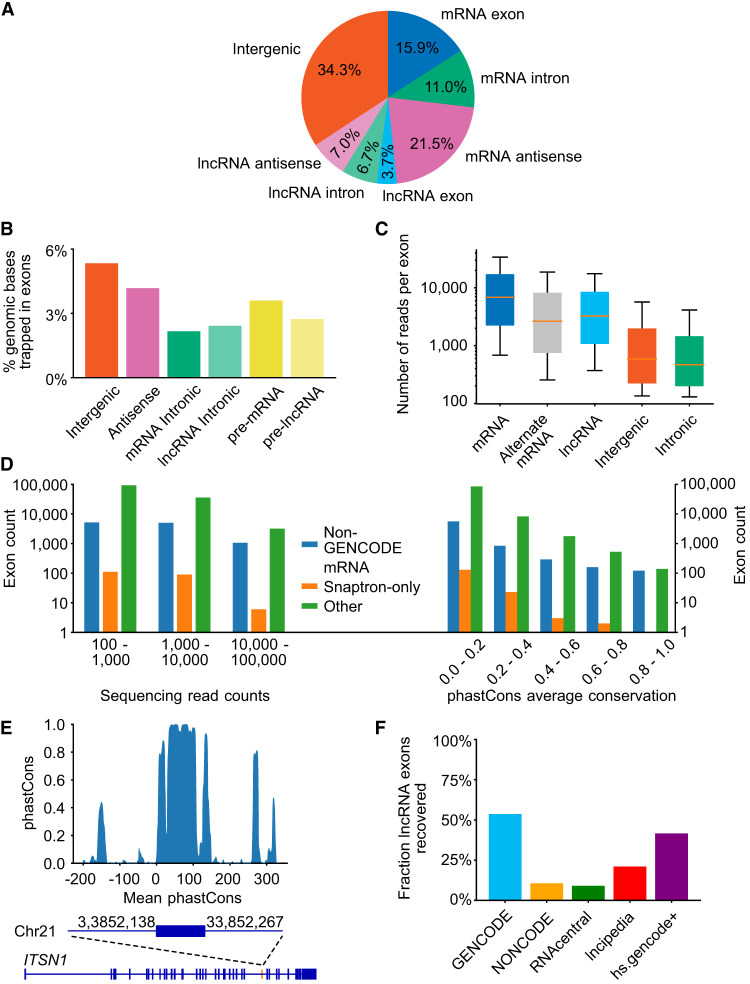
Trapped exons found in different categories of genomic region. (*A*) Pie chart depicting the proportions of exons from different genomic regions. The percentage of exons for indicated genomic regions relative to the total read count is indicated. (*B*) Bar plots showing the percent of genomic bases in a trapped exon for different categories of genomic region. (*C*) Boxplot depicting trapped exon sequencing read counts for different genomic regions. Whiskers depict 10th and 90th percentiles. (*D*) Bar plots depicting the exon counts at different read count bins (*left*) or average phastCons score bins (*right*) for different non-GENCODE v37 exon annotations. Exon counts are shown with logarithmic scale. Non-GENCODE v37 exon annotations, Snaptron database, and exons found by this exon trapping study are displayed. Average phastCons scores were calculated using the sequence of the exons. (*E*) Bar plot depicting phastCons (30-way) scores for +/− 200 bp around an unannotated sense intronic exon found by exon trapping in gene *ITSN1.* (*F*) Proportion of annotated exons recovered from various databases. Blue bar indicates trapped exons that are annotated in GENCODE mRNA/lncRNAs. Trapped exons annotated in other lncRNA databases are shown, with annotated GENCODE lncRNAs removed.

We next categorized the ∼1.25 million trapped exons relative to known gene features. Figure 3A shows the proportion of exons that overlap major categories of genomic sequence annotation. The largest fraction of trapped exons is “intergenic,” followed by mRNA antisense, likely because of the fact that these sequences represent most of the genome: Per base, only a small fraction of each is trapped (Fig. 3B). For example, 5.3% of all intergenic region bases (i.e., excluding any kind of mRNA or lncRNA, and their antisense sequence) are part of a trapped exon, corresponding to an exon every 3311 bases on average. Because there is a large amount of intergenic sequence, the absolute number of “intergenic” exons is high (424,632). Similar proportions, and corresponding numbers of exons, are obtained for antisense strands (Fig. 3B).

A large proportion of the trapped exons (11.0%) was found within mRNA introns, in the forward strand (Fig. 3A), but the fraction of intronic sequence encompassed is lower than it is for other regions, particularly the mRNA antisense strand (Fig. 3B). This outcome is consistent with selection against fortuitous exon-like sequences within introns; that is, exons that arise by chance in the antisense orientation are inconsequential, whereas exons that arise by chance in the sense orientation (i.e., within introns of coding pre-mRNAs) will be deleterious, and thus removed over time. These sequences nonetheless exist, and could represent potential alternative exons, or regulatory exons that trigger NMD. It is also conceivable that they are excluded by context-specific mechanisms (e.g., the sequences of neighboring exons) that are not present in our library plasmids. The “intronic” trapped exons also displayed lower overall inclusion rates than any other category, including “intergenic” exons (Fig. 3C). Many of the “intronic” exons, especially those with higher inclusion levels, are found in other mRNA databases (but not GENCODE) (6985); an additional 240 are found in mRNA-seq data (from the Snaptron database [Wilks et al. 2018]), indicating that they are used in their genomic context (Fig. 3D, left). In addition, a subset of the “intronic” exons displays primary sequence conservation (Fig. 3D, right). Figure 3E shows an unannotated region from the *ITSN1* gene which is both conserved and trapped at high levels (10,917 read counts).

Known alternative cassette exons displayed, on average, 2.5-fold lower inclusion rates when compared to all internal mRNA exons (Fig. 3C). First and last (i.e., terminal) mRNA exons, however, which would be expected to lack either the 3′SSs or the 5′SSs, respectively, were rarely captured: Only ∼4% of these are present among the trapped exons, consistent with the rate of fortuitous splice sites across the genome.

Notably, 52% of GENCODE lncRNA internal exons were trapped with at least 100 reads (Fig. 3F), a figure comparable to that of mRNA internal exons (61%). The recovery of lncRNA internal exons that are only present in the other lncRNA databases (and not GENCODE), however, averages only 9.6% for the four databases interrogated (Fig. 3F; Supplemental Fig. S5), suggesting that these exons may have lower splicing efficiency. We assume that the well-curated GENCODE data set is enriched for lncRNAs that splice efficiently, relative to lncRNAs that are only present in the other databases, because the impact on RNA abundance leads to a higher curation rate.

### Sequence features of trapped exons

**Figure 4.**
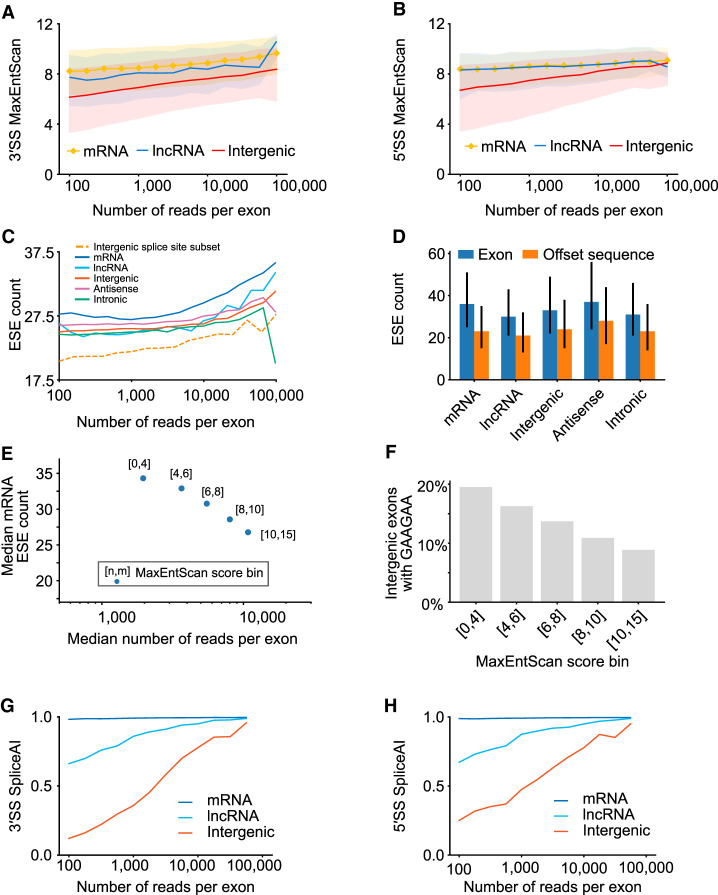
Known splicing signal correlations with exon read counts. (*A*) Line plots depicting 3′ MaxEntScan scores for trapped exons from different genomic regions. Exons from mRNA, lncRNA, and Intergenic regions are indicated and are binned by logarithmic read counts. Median values are displayed as lines with shaded region corresponding to 25th–75th percentiles. (*B*) Same as *A*, above, but depicting 5′ MaxEntScan scores. (*C*) Line plots representing Splicing Enhancer (ESE) counts for trapped exon sequences from different genomic regions. ESE median values are displayed, and exons are binned by their logarithmic sequencing read counts using logarithmic bins ranging from 100 to 10,000. (*D*) Bar plots depicting the median ESE counts for trapped exons (blue bars) and nearby sequence of the same length offset by 250 bp (orange bars) for different genomic regions. *Offset* sequences are the same length as the associated exon and correspond to coordinates 250 bp upstream for reverse strand exons and 250 bp downstream for forward strand exons. For forward strand exons this is downstream from the exon and for reverse strand exons this is upstream of the exon. Range lines indicate 25th–75th percentiles. (*E*) Scatter plot representing ESE count (*y*-axis) versus median sequencing read count (*x*-axis) for trapped exons, subdivided by MaxEntScan scores into groups with weaker to stronger splice sites based on splice site score bin (point label). Splice site bins indicate that contained exons have both their 3′SS and 5′SS splice sites within the labeled MaxEntScan score boundaries, between values indicated by [n,m], where n = lower score and m = upper score. (*F*) Bar plot depicting fraction of intergenic exons that contain the ESE GAAGAA nucleotide sequence. Individual bars correspond to exons with both 3′SS and 5′SS MaxEntScan scores (see *E*, above) within the range given in the bar label (e.g., [n < splice site MaxEntScan score < m] for both 3′SS and 5′SS MaxEntScan scores). (*G*) Line plots depicting 3′SS SpliceAI scores for trapped exons in different genomic regions. Values in the *x*-axis are logarithmic sequencing read counts using bins from 100 to 10,000 with 25 steps. For intergenic exons, the Spearman's correlation between SpliceAI scores and read counts is 0.31. (*H*) Same as *G*, above, except for 5′SS SpliceAI scores. For intergenic exons, the Spearman's correlation between SpliceAI scores and read counts is 0.12.

We next examined whether unannotated exons in the exon trapping data set contained known sequence features of exons. We first considered the splice site scores, using MaxEntScan, which outputs a maximum entropy-based score indicating whether a given base location is a 5′ or 3′ splice site (Yeo and Burge 2004). Read counts per exon displayed a positive overall correlation with MaxEntScan scores, for mRNA, lncRNA, and “intergenic” exons (Fig. 4A,B). The MaxEntScan scores of the intergenic exons display a wider spread, however, and a lower (albeit overlapping) score distribution to the annotated mRNA and lncRNA exons, for identical read counts.

We also investigated the prevalence of known splicing enhancing sequences within exonic regions, focusing on potential general splicing enhancer (ESE) hexamers from Ke et al. (2011). We observed that the frequency of ESEs increases with the exon read count and that this relationship is more prominent when exons are subset by their splice site scores (Fig. 4C; Supplemental Fig. S6A). We reasoned that strong ESEs may offset weak splice sites, and consistent with this notion, the relationship between ESE count and read count becomes more prominent when the splice site scores are subset to narrow ranges (e.g., Fig. 4A; Supplemental Fig. S6B). The number of ESEs within exons also contrasts with surrounding sequence (Fig. 4D), illustrating that the ESE enrichment is not simply a feature of local genomic sequence. Moreover, exons with lower MaxEntScan scores have higher ESE density, on average (Fig. 4E; Supplemental Fig. S6B), further indicating that ESEs play a role in exon identity. There is a particularly strong trend for the individual SR protein-binding ESE hexamer GAAGAA (Fig. 4F; Fairbrother et al. 2002), which is present more than twice as often in intergenic exons with the weakest splice sites versus strongest splice sites (20% vs. 8%).

We also considered splice site scores from SpliceAI (Jaganathan et al. 2019), which should recognize both splice site strength and the presence of other sequences that impact splicing. The median SpliceAI prediction scores correlate most strongly with exon trapping inclusion rate for “intergenic” exons, which were not part of the SpliceAI training data (Fig. 4G,H). The mRNA exons, used in training SpliceAI, typically have maximal SpliceAI scores, irrespective of their autonomous splicing potential (i.e., read count), suggesting that SpliceAI has learned features of mRNA exons that are distinct from their autonomous splicing potential. Average SpliceAI scores for GENCODE lncRNA exons are higher than those of intergenic exons, but lower than those of mRNAs exons, for equivalent read counts (Fig. 4G,H), consistent with the notion that the additional features learned by SpliceAI do not relate only to coding potential, and may encompass productive elongation or transcript stability (Jaganathan et al. 2019).

### Many trapped exons lack known exon-associated sequence features

**Figure 5.**
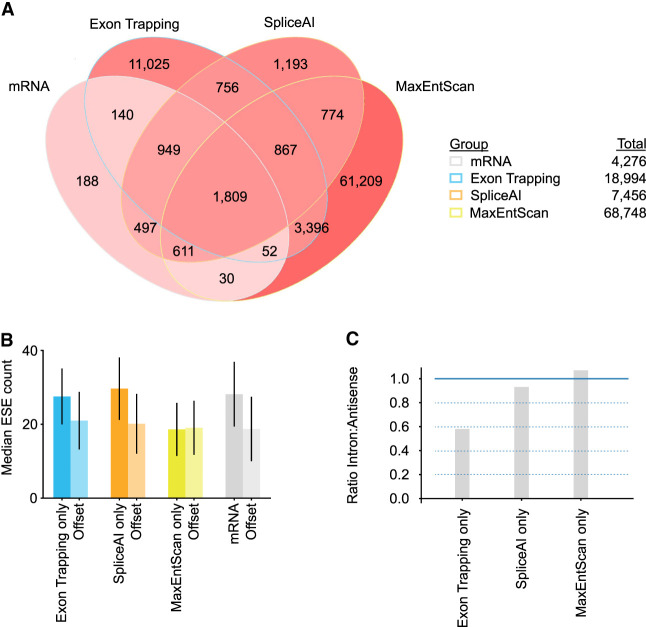
Overlaps between exons detected using different approaches. (*A*) Venn diagram depicting Chromosome 17 forward strand exons found by different exon calling approaches. Exons are labeled as mRNA (annotated mRNA & lncRNA internal exons), ET (exons found by exon trapping), MaxEntScan (based on MaxEntScan scoring), and SpliceAI (based on SpliceAI scoring). Exon counts corresponding to overlapping regions are indicated and are colored red linearly with intensity determined by (log_10_ #exons). (*B*) Bar plot of median ESE counts for exons and offset sequences identified using approaches listed in *A*. *Offset* sequences are the same length as the associated exon and correspond to coordinates 500 bp upstream for reverse strand exons and 500 bp downstream for forward strand exons. For forward strand exons this is downstream from the exon and for reverse strand exons this is upstream of the exon. Range lines indicate 25th–75th percentiles. Refer to *A* for *x*-axis labels. (*C*) Bar plots showing the ratio of intronic to antisense exon counts found for the different exon finder approaches.

We next asked whether the sequence features above could completely account for the trapped exons. To do this, we required exon predictions that are based only on these sequence features. Neither SpliceAI nor MaxEntScan explicitly predict exons, but it is possible to derive exon predictions by simply associating strong predicted 3′ splice sites with proximal strong predicted 5′ splice sites (here, using a SpliceAI score cutoff of 0.2, a MaxEntScan score cutoff of 6, separated by 63–222 bases [10%–90% percentile of mRNA internal exons]). We examined the overlap of such exons predicted across Chromosome 17 (to reduce computation time) from either the SpliceAI or MaxEntScan outputs, and compared to exonic regions we obtained from exon trapping, and annotated exons. We separately tallied exons that overlapped perfectly (Fig. 5A) from those that overlapped partially (Supplemental Fig. S7A).

As described above, most of the annotated exons were captured by exon trapping, but a large proportion of the trapped exons did not overlap with annotated exons. The majority of trapped exons also did not overlap with exons predicted by either SpliceAI or MaxEntScan: more than half of the trapped exons—11,025/18,994—did not overlap with any of the other exon sets. We asked whether these 11,025 trapped exons have characteristic properties beyond their low 5′ or 3′SS scores (which we infer because they were not detected by MaxEntScan) (Supplemental Fig. S7B,C). Their lengths and base content are not unusual (Supplemental Fig. S7D,E). They overlap with repetitive elements at roughly the frequency (∼50%) that repetitive elements occur in the genome. The trapped exons are enriched for ESEs, however, relative to adjacent sequence, to a degree that is similar to known exons and SpliceAI-predicted exons (Fig. 5B). The difference in ESE frequency appears insufficient to explain why these exons are included, however: The range largely overlaps with that of the tens of thousands of MaxEntScan-predicted exons, which have stronger splice sites overall, and yet are not trapped. We also asked whether the detection of these exons might be because of their genomic context. To do this, we queried the density of in situ sequences (ISS) (Wen et al. 2010) for mRNA and trapped intergenic exons, reasoning that there may be ISS sequences in distal intergenic genomic sequence (and not mRNA introns) that would be absent from the reporter inserts. We observed little difference, however (Supplemental Fig. S8A,B).

An additional and intriguing observation is that, as noted above, the trapped exons are very significantly depleted from introns (*P* < 7.1 × 10^−293^, Wilcoxon rank-sum test), but not the mRNA antisense strand, and trapped exons that are detected in introns tend to have low read counts (median 448 vs. 6214 for mRNA). Among exons detected only by exon trapping, there is a twofold bias toward introns (Fig. 5C). In contrast, exons predicted only by SpliceAI or MaxEntScan have roughly similar numbers of exons within annotated introns, relative to exons predicted in the antisense strand (Fig. 5C). Altogether, we conclude that the exon trapping data must contain some biologically meaningful information not captured by the splicing predictors.

### Transposons as a source of novel exons

**Figure 6.**
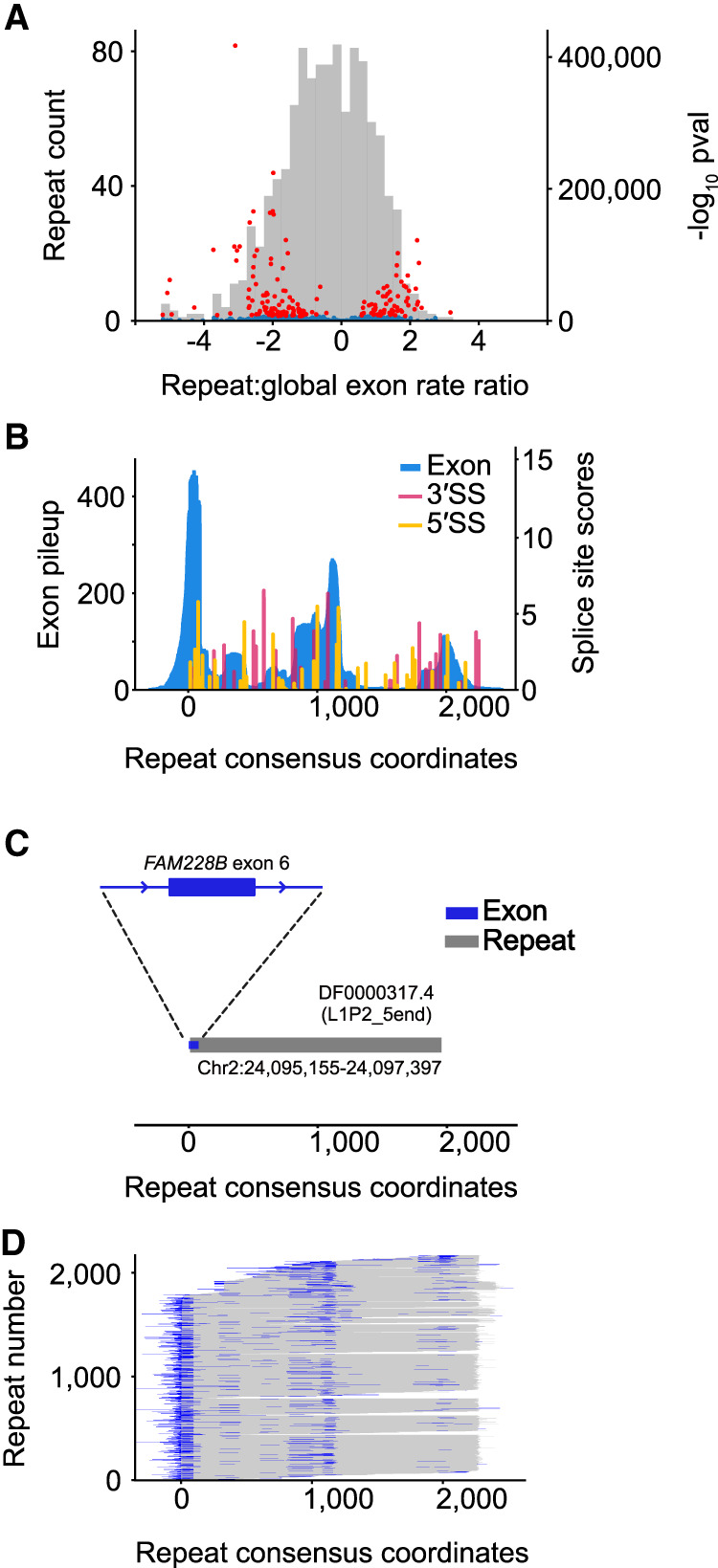
Exons overlapping repetitive elements. (*A*) Histogram depicts ratio of exon overlaps for different repeat families, relative to the global genomic exon rate. Volcano plot shows log_10_ *P*-values of repeat family exon bases enrichment (hypergeometric test) versus the repeat exon enrichment relative to the global exon rate. Red dots indicate top 5% of *P*-values. Blue dots indicate the bottom 95% of repeat-values. (*B*) Histogram pileup depicting sequencing reads overlapping repeat instances of DF0000317.4 (5′ end of L1 retrotransposon L1P2) in the human genome. Histogram maps sequencing reads in the genome to the Dfam repeat consensus model. MaxEntScan 3′SS and 5′SS scores are also shown across the Dfam repeat consensus model with scores above 0 shown with colored bars. Repeat consensus coordinates start at 0. (*C*) Diagram depicting overlap between trapped exon 6 from *FAM228B* (Chr 2: 24,095,141–24,095,230) and a genome instance of L1P2 transposon DF0000317.4 (Chr 2: 24,095,155–24,097,397). (*D*) Diagram depicting overlap between all trapped exons and associated genomic repeat instances for L1P2 transposon DF0000317.4. Rows are sorted by repeat start and end coordinates for the Dfam repeat consensus model.

Transposable elements (TEs) make up roughly half of the human genome and are a prevalent source of new genetic material, including exons (Sorek 2007). *Alu* elements, for example, are a source of alternative mRNA exons because of the presence of sequences consistent with splice sites near the repeat's 5′ end and an upstream polypyrimidine tract arising as a product of retrotransposition (Makalowski et al. 1994; Dewannieux and Heidmann 2005; Sorek 2007). We asked whether specific classes of TEs are enriched or depleted among the trapped exons, on a base-by-base level, and found many cases of both enrichment and depletion (Fig. 6A). We reasoned that enrichment of specific TE classes might be because of splice sites within the TE, and indeed such cases are readily identified. Examination of compiled instances across the genome, and inspection of the consensus models on Dfam 3.4 (Storer et al. 2021), revealed that the trapped exon often corresponds to a common segment of the transposon, beginning and/or ending at a location where the ancestral element contained sequences resembling 5′ and/or 3′ splice sites. An example (DF0000317.4, the 5′ end of L1 retrotransposon L1P2) is shown in Figure 6B. This repeat overlaps four annotated mRNA exons; one example is depicted in Figure 6C. The ∼2000 additional trapped exons overlapping this repeat largely favor internal exons arising inside near full-length repeat sequences (Fig. 6D). We assume that these sequences are fortuitous, because splice signals are short and degenerate, but they nonetheless represent a ready means by which these transposons can contribute to the evolution of existing genes.

### Conservation of splice sites and trapped exons

**Figure 7.**
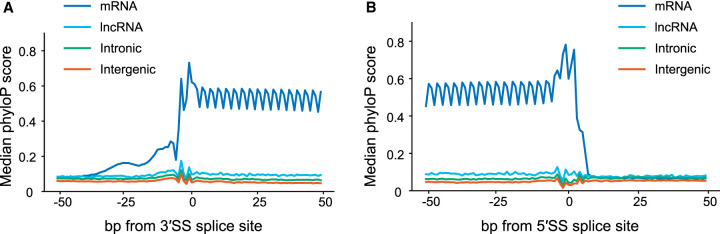
Conservation around 3′ and 5′ splice sites of trapped exons. (*A*) Diagram depicting sequence conservation of trapped exons around 3′SS for different genomic regions. Shown are phyloP scores for the 100 bp region centered around the 3′SS for trapped exons from mRNA, lncRNA, and intergenic regions, as indicated. (*B*) Same as *A* except phyloP scores were calculated for the 100 bp region centered around the 5′SS for trapped exons.

Finally, we examined trends in sequence constraint of various types of exons: coding exons, trapped exons found in annotated introns (“intronic”), lncRNA exons, and novel trapped exons found in intergenic regions (“intergenic”). Figure 7, A and B, shows that the splicing sequences of known coding exons are highly constrained (by phyloP [Pollard et al. 2010]), and also shows a three-base periodicity within the exons, presumably because of codon bias and wobble. In contrast, none of the other classes displayed strong conservation, on average. For lncRNAs this phenomenon is well-documented (Wang et al. 2004a; Kutter et al. 2012; Li and Yang 2017). The intergenic regions detected by exon trapping therefore behave much like lncRNA exons in this aspect.

## Discussion

An exon trapping screen of the human genome in a single cell type captured >1 million sequences that splice as internal exons. Most known mRNA exons are trapped, demonstrating that exons of coding genes are predominantly autonomous, consistent with the exon definition model. Moreover, splicing-competent sequences are common in nongenic locations of the human genome. This finding presents a ready explanation for the large number of poorly conserved lncRNAs.

A central finding of these exon trapping assays is that a majority of the known exons in human protein-coding genes are autonomous. This observation does not rule out a role for context (i.e., flanking sequence) in exon recognition. A clear limitation is that only five different splicing vectors were used, and they are highly related to each other. The assay in its current form was not intended as a universal exon discovery tool, only as an initial survey. The fact that most coding exons were trapped, however, indicates that a specific context is not required for splicing of most individual exons. Another limitation of this study is that the screens were performed in a single cell type. Thus, the impact of cell type–specific splicing factors would not have been captured. To our knowledge, such factors would mainly be expected to influence alternative exons, which were indeed trapped at lower rates.

The data also show that there are many sequences in intergenic regions, and antisense to known genes, that can be spliced into a heterologous transcript. Such sequences also exist in introns, but at a significantly reduced frequency. These “intronic exons” bear a resemblance to pseudoexons—intronic sequences that are flanked by splice sites but that are not observed in spliced mRNA. Aberrant inclusion of pseudoexons is thought to represent an underreported disease mechanism (Petersen et al. 2022). These exons appear to be determined in part by the simple presence of strong splice sites in close proximity, but there are many which have weak splice sites. Frequency of ESE sequences provides only a partial explanation for their inclusion as exons. Given the large number of variables to explore (e.g., positioning and relative weights among ESEs; presence of intronic cues; splicing silencers; and combinations or conditional relationships among features), we did not attempt to develop new exon predictors as part of this study. Nonetheless, the data presented here, which includes not only many new autonomous exons, but also associated read counts, provides a new resource for analysis of both exon identity and exon inclusion levels spanning a large dynamic range.

It is possible that the hundreds of thousands of exons detected by exon trapping in intergenic and antisense regions are not already annotated as exons mainly because they are not expressed, and/or do not form part of stable transcripts. Moreover, the observation that autonomous exons with little or no sequence constraint occur frequently in intergenic space suggests that the required sequence features arise frequently by random mutations. SpliceAI, which was trained to predict splice sites in coding exons, also readily predicts splice sites in intergenic sequence (Fig. 5). SpliceAI also predicts splice sites in random sequence at similar rates to what is observed in the exon trapping data: Using the thresholds described earlier, it identifies 1856 exons in a dinucleotide-permuted positive strand of Chromosome 17. This number, and the range of splice site scores, is similar to the number of unannotated exons found on the real positive strand Chromosome 17 (Supplemental Fig. S7F). This outcome is consistent with the low information content of splice sites, and the fact that nearly 25% of 6-mers show ESE activity (Ke et al. 2011).

We speculate that lncRNAs, whose promoters often overlap with endogenous retroviral LTRs and enhancers (Kelley and Rinn 2012; Engreitz et al. 2016), may result from coupling promoter-like sequences to randomly arising autonomous exons in the surrounding DNA. This notion is consistent with the fact that there is little overall evidence of selection on lncRNA primary sequence (Wang et al. 2004a). In addition, the read counts we obtained for lncRNA exons are only about half of those obtained for coding exons, and similar to the read count abundances of alternative exons. Lower splicing efficiency could partly explain the lower expression levels and lower exon numbers of lncRNAs relative to mRNAs (Orom et al. 2010) and is consistent with observations that lncRNA exons are often chaotically spliced (Deveson et al. 2018).

These findings reinforce speculation that much of the “dark matter” transcriptome may be a by-product, or even an expected component, of the regulation of known genes, as well as a source of novel genetic entities. The exon trapping data presented here identify regions that would be incorporated into such transcripts, if expressed. These data also offer an orthogonal set of genomic exons appropriate for understanding splicing sequences not under mRNA selection, providing sequences across a large dynamic range that should enable additional insights into the splicing code.

## Methods

### Reporter intron

We gene synthesized the 6th intron (Chr 3: 185,919,497–185,921,103, “–” strand) of the gene *TRA2B* as the reporter intron. This intron has strong splice sites and a native length of 1.6 kb. We changed several bases to remove possibly cryptic 5′SS splice sites and added sequence to the middle of the intron to facilitate genomic fragment insertion by restriction digest and Gibson assembly.

### Plasmid preparation

The five different plasmid backbones (Supplemental Details sheet “Vectors,” Supplemental Vector details) were generated as follows. The three plasmids that vary in reading frame position of the splice site were designed so that the splice sites are compatible with the coding sequence; these were ordered by gene synthesis and cloned by Gateway into a pcDNA3.1-based plasmid. The two variants with mutations in the 5′SS and putative RBFOX1 binding site, respectively, were made using Gibson assembly and primers containing variant sequences (see Supplemental Details; Fig. S1A).

To generate libraries, each of the five backbones was inoculated in *Escherichia coli* (ElectroMAX Stbl4 competent cells) and plasmid DNA was extracted using Qiagen HiSpeed Midiprep kit. The plasmids were digested with restriction enzymes as follows. 10 µg plasmid was incubated in 250 µL 1× CutSmart buffer with 10 µL AgeI-HF enzyme and 10 µL NotI-HF enzyme, at 37°C for 20 min. DNA was then purified using Zymo Clean and Concentrator 5 at 2:1 binding buffer:restriction digest. DNA was eluted twice, incubating for 1 min with 70°C 8 µL of NEB DNA elution buffer. (For reagents, see Supplemental Details sheet “Products.”)

We prepared two different adapter duplexes (age_25/age_common and not_25/not_common, Supplemental Details sheet “Oligos”), one for the AgeI site and one for NotI by heating 10 mM of the oligo pair in 25 mM NaCl + 0.5 mM EDTA to 90°C for 2 min moving to room temperature for 5 min.

### Adapter-ligated gDNA preparation

We extracted gDNA from HEK293 cell cultures (six well plate) using the PureLink Genomic DNA Mini Kit, and fragmented 10 µg of the resulting DNA using NEB NEBNext dsDNA Fragmentase (1.6 mg of gDNA in 54 µL of 1× dsDNA Fragmentase buffer, incubate on ice for 5 min, add 6 µL dsDNA Fragmentase, mix by vortexing, incubate at 37°C for 20 min, stop with 15 µL 0.5M EDTA mixed by pipetting). The resulting fragmented DNA was purified with Zymo Clean and Concentrator 5 µg and eluted with NEB DNA elution buffer. We then end repaired the DNA using NEB end repair kit and added dA tails using NEB dA-tailing. We then ligated the eluted gDNA (20 ng/µL reaction volume) to dT-tailed DNA adapters (generated by annealing two oligos pairs (age_25/age_common and not_25/not_common, Supplemental Details sheet “Oligos”) for 30 min at room temperature using NEB Quick Ligase. We cleaned the reactions using 1.8× AMPure XP beads and eluted the DNA with NEB elution buffer supplemented with Tween 20 to 0.1%.

DNA was then amplified by PCR using NEB Q5 enzyme and primers complimentary to the adapters (NEBb_1, NEBb_2, Supplemental Details sheet “Oligos”) (PCR program 98°C 30 sec; 98°C 10 sec, 57°C 15 sec, 72°C 60 sec [N times]; 72°C 120 sec). At this and subsequent PCR steps, we performed qPCR to determine the number of cycles to amplify the library. We used the Q5 HF protocol scaled to 60 µL with 1 µL of cDNA, dispensed as three replicates of 15 µL, 1× Evagreen dye, 0.5 mM primer. We selected the cycle number that is two cycles before 50% amplification is exceeded.

DNA was sized on 1% agarose gel cast with 1× SYBR Safe to a range of ∼500−1100 bp using Quick-Load Purple 100 bp DNA Ladder. DNA was gel extracted using Qiagen QIAEX II Gel Extraction Kit, and eluted with 25 µL of NEB DNA elution buffer. Eluted DNA was again amplified, in preparation for Gibson assembly, with the number of PCR cycles determined by qPCR to avoid saturation.

### Gibson assembly and library amplification

Gibson assembly of gDNA into plasmids was performed using NEBuilder HiFi DNA Assembly Master Mix in 200 µL with 2 µg plasmid and 300 ng of gDNA. The five assembled plasmid libraries were phenol extracted and precipitated, and DNA was resuspended in 12 µL NEB DNA elution buffer. Resuspended DNA was electroporated using 2 µL plasmid and 20 µL of ElectroMAX Stbl4 competent cells according to kit protocol, with five replicates for each of the five plasmid libraries. *E. coli* transformants were grown overnight with shaking at 30°C in 110 mL of LB with Carbenicillin antibiotic (100 mg/mL).

Overnight cultures were split into 2 × 50 mL and pelleted in a tabletop centrifuge set to 4°C, supernatant discarded, and pellets stored at −20°C. Cell pellets were extracted using Qiagen HiSpeed Midiprep kit and eluted using 500 µL of the supplied TE buffer.

### Transfections

We plated 1.2 million HEK293 cells into 10 cm plates and incubated overnight in DMEM to ∼75% confluency at the time of transient transfection. We performed transient transfections with 10 µg of plasmid DNA using Promega ViaFect according to reagent protocol. DNA was incubated with the ViaFect transfection reagent and Opti-MEM for 15 min at room temperature before transfection. The growth medium was changed after 24 h, and after a further 24 h, the transfected cells were harvested from each plate with 10 mL of TRIzol, and stored at −80°C.

### RNA extraction

We thawed the TRIzol-cell mixture by incubating on ice for 10 min. Two (2) mL of chloroform was added and mixed by inversion, then tubes were incubated at room temperature for 2 min.

We then centrifuged the tubes for 15 min at ∼4000 × *g* at 4°C. We split each sample's aqueous phase into two pre-spun phaselock tubes (pre-spun with 1 mL of BCP). We added an equal volume of acid phenol (∼3 mL) and 0.2 volume of BCP (∼0.6 mL) to each tube, mixed by inversion, and incubated at room temperature for 2–3 min before centrifuging for 15 min at ∼4000 × *g* at 4°C. We performed a final spin by adding 1 mL of chloroform to each sample which we mixed by inversion, incubated at room temperature for 2–3 min, and centrifuged for 15 min at ∼4000 × *g* at 4°C. We transferred the supernatant.

We precipitated these samples using sodium acetate ethanol precipitation along with 10 µL of Glycoblue coprecipitant. Precipitated RNA was resuspended in 300 µL of H_2_O.

### Poly(A) enrichment

We isolated poly(A) RNA using the Invitrogen Poly(A) Purist MAG Kit. Each sample of extracted RNA was brought to 500 µL with 200 µL H_2_O. We then added 500 µL of 2× binding buffer. This mixture was added to 30 µL of prepared beads. This mixture was denatured at 75°C while shaking at 300 rpm. Samples were eluted twice, using elution buffer heated to 80°C. 2 µL glycogen was added to the poly(A) RNA and samples were precipitated per kit instructions. Precipitated RNA was resuspended in 40 µL of THE RNA Storage Solution (Invitrogen).

### Reverse transcription

We reverse transcribed the RNA using SuperScript IV and a primer targeting reporter transcript sequence downstream from the 3′ exon (pTH_T5_RT_3). We followed the kit protocol scaled to 3.5× volume with 2 mM of pTH_T5_RT_3 and 38.5 µL of Template RNA. cDNA synthesis was performed for 11 min. We hydrolyzed RNA using the cDNA clean-up protocol from Zymo DNA Clean Concentrator-5 with volumes scaled 1.4-fold. We performed the final elution with 20 µL of NEB DNA elution buffer warmed to 65°C.

### Amplification with reporter-specific primers

We PCR amplified the reporter transcript with NEB Q5 polymerase and primers targeting the flanking exons, tailed to add Illumina Nextera sequences (primer pairs NS601–NS602, NS603–NS604, NS605–NS606) (PCR program 98°C 30 sec; 98°C 10 sec, 70°C 15 sec, 72°C 60 sec [N times, determined by qPCR]; 72°C 120 sec), where we determined the number of cycles, N, to amplify using qPCR as described previously.

### PCR 1: sizing using native acrylamide gels

We cleaned the PCR with Ampure XP beads. We gel-sized this PCR reaction using Bio-Rad precast 5% Mini-PROTEAN TBE Gels run for 45 min at 75V in 1× TBE buffer. We stained the gels using 1× SYBR Gold and extracted all material longer than the empty reporter PCR product (i.e., ∼ to the top of the well). We eluted and recovered the DNA from the gels slice using QIAEX II Gel Extraction Kit, using the kit protocol for native acrylamide gel extraction. We eluted the DNA in diffusion buffer at 50°C and 1400 rpm for 1 h, followed by overnight rotation at room temperature. During the subsequent recovery of DNA from the diffusion buffer, DNA was incubated with shaking (1400 rpm) for 10 min, instead of occasional vortexing. The recovered DNA was eluted using 25 µL of NEB elution buffer supplemented to 0.1% Tween 20.

### PCR 2: amplify DNA with Illumina index primers

We amplified the gel-sized DNA in order to attach index sequences, and the remaining sequences required for Illumina sequencing. This PCR used Illumina UDI primers (PCR program 98°C 30 sec; 98°C 10 sec, 70°C 15 sec, 72°C 60 sec [N times, N determined by qPCR]; 72°C 120 sec). Index usage in Supplemental Details sheet “Indexes” and “Oligos”. We determined the number of cycles to amplify using qPCR as described previously. We then cleaned and sized the PCR again as described above.

### PCR 3: amplify DNA for sequencing

We PCR amplified the sized DNA using oligos corresponding to Illumina P5 and P7 sequences (PCR program 98°C 30 sec; 98°C 10 sec, 70°C 15 sec, 72°C 60 sec [N times]; 72°C 120 sec). We determined the number of cycles to amplify using qPCR as described previously. We cleaned these PCRs using Ampure XP beads and eluted using 20 µL NEB DNA elution buffer supplemented to 0.1% with Tween 20.

### Illumina sequencing

Sequencing was performed on an Illumina NovaSeq S4 flowcell, with 2 × 150 bp reads. The sequencing facility (The Centre for Applied Genomics and The Hospital for Sick Children, Toronto) performed Ampure XP clean up on some samples, to reduce Illumina dimers, and “dark cycled” (chemistry only) the first 18 bases of read 1, in order to bypass constant sequences of the reporter assay, and increase sequencing read base complexity used for Illumina flowcell deconvolution processing. The dark cycling encompasses part of the 3′ splice site; thus, 3 or 4 bases of each exon are removed (because different length primers were used, depending on backbone).

### Illumina data processing and read mapping

We truncated both read 1 and read 2 to the first 45 bases, and removed reads with cigar strings containing “S”. We used BBDUK to trim adapter sequences and remove empty reads. BBDUK was run with additional parameters “ktrim = r k = 13 mink = 5 hdist = 1 tpe tbo threads = 2”.

We aligned trimmed sequencing reads to the hg38.2bit genome sourced from UCSC (https://hgdownload.cse.ucsc.edu/goldenpath/hg38/bigZips/hg38.2bit). Alignments were performed using HISAT2 (Kim et al. 2015) with parameter “‐‐max-intronlen 1500”.

To aggregate reads, we sorted the individual aligned libraries by their chromosome position (chromosome, 5′ coordinate and then 3′ coordinate, then strand), using linux sort with C locale. We retained mapped reads with mapping quality greater than or equal to 20. We adjusted the mapped read termini to account for the 3–4 bases of the 5′ exon end that were clipped by the “dark cycle” sequencing (library adjustment in and Supplemental Details sheet “Indexes”). Reads with identical ends were counted and collapsed into one entry in a new file, which represents exon read counts. Most of the analyses in the paper were performed using data pooled from the different libraries, which were merged by collapsing the results from the five libraries using the same overall approach. To account for sequencing indels, we also collapsed overlapping exons (i.e., groups of overlapping reads) by first adjusting the termini of all exons up to two bases, if the adjustment coincides with a higher scoring splice site, and then again collapsing these reads to arrive at the final exon data set.

### Removal of exons associated with the reporter intron

Some of the most abundant exons align to the native reporter intron sequence which is present in every assayed plasmid. We removed exons that are fully contained in the *TRA2B* intron we used (Chr 3: 185,919,497–185,921,103) because these counts are presumably rarely splicing exons in the reporter present in every assayed plasmid.

### GENCODE gene annotation

We used GENCODE v37 “basic” to determine annotated exons. Transcripts with any “transcript support level” were included in analyses. Transcripts with “level” 3 or less were included for analysis. The mRNA exons are GENCODE exons of gene_type=“protein_coding”. The lncRNA exons are GENCODE exons of gene_type=“lncRNA”. First and last exons were obtained for each mRNA transcript. The remaining exons of each transcript define the internal mRNA exons. The GENCODE lncRNA exons were obtained in the same manner. Transcript boundaries were obtained from the first and last exon coordinates.

We aggregated all remaining exons into a total exon trapping data set. We created a primary exon data set by grouping overlapping exons sharing a 3′SS. Primary 3′SS clusters with greater than or equal to 100 sequencing reads were used to threshold exon trapping exons. The exon with the highest number of sequencing reads was selected as the primary exon to represent this exonic region. For exons sharing the same 3′SS we selected the exon with the majority of reads as the primary exon.

### Intergenic, intronic, and antisense exons and region categories

We defined the intergenic regions as those that do not overlap with sense mRNA/lncRNA transcripts or their antisense regions. Intronic regions were identified in transcript regions with all annotated exons removed. We selected the antisense regions by starting with the antisense regions of transcripts and removing any annotated transcript (this, for example, excludes annotated antisense transcripts). We determined the exon trapping exons of each region type by taking those exons fully contained within the above-described regions.

### Conservation

We used hg38 phyloP 7way sourced from UCSC for phyloP conservation scores (https://hgdownload.cse.ucsc.edu/goldenPath/hg38/phyloP7way/). phastCons scores were computed using the hg38 30way file (https://hgdownload.soe.ucsc.edu/goldenPath/hg38/phastCons30way/).

### Alternative exons

We used the database HEXEvent to select alternative exons. We downloaded the data using the associated website (http://hexevent.mmg.uci.edu/HEXEvent/example.html) for all chromosomes and those exons of type cassette or constitutive. We called an exon alternative if the constitLevel score was ≤0.9. We called exons constitutive if the constitLevel score ≥0.99.

### Repetitive elements

We used Dfam v3.4 repeat annotations. We used both the coordinates of the consensus models (https://dfam.org/releases/Dfam_3.4/families/Dfam.embl.gz) and tallied exons overlapping the Dfam genome hits (https://dfam.org/releases/Dfam_3.4/annotations/hg38/hg38_dfam.nrph.hits.gz) along the coordinates of the consensus models. We analyzed repeats overlapping an exon with a read count of 100 or greater.

We calculated log *P*-values for the enrichment of exons in each repeat using the hypergeometric test, computed using the Python SciPy function logpmf (with M = number of stranded genome bases, n = number of exon bases, N = the number of genome bases for the repeat family tested, k = the number of exon bases overlapping the repeat family tested).

### Exonic splicing enhancer database

We used the hexmer sequences splicing enhancers (Ke et al. 2011), which yielded 1182 hexmer exonic splicing enhancers out of a possible 4096 hexmers. These splicing enhancers sequences were used to count the occurrences of ESEs in the queried exon sequences. ESE counts were normalized to the number of ESE per 100 bp of exon sequence.

### SpliceAI scoring

We obtained the SpliceAI models by installing the Python SpliceAI module and locating the trained model files. The models were loaded using the tensorflow Keras submodule. We obtained the average score of the five models for the query DNA sequence. The five-model average score was used as the SpliceAI score for a given exon. When comparing mRNA, lncRNA, and intergenic exon SpliceAI scores, the scored sequence was the genomic sequence from 500 bp upstream of and downstream from the exons 3′SS and 5′SS for up to 10,000 exons. This sequence was padded with “N” characters for the remainder of the scoring window. Only exons that do not overlap repetitive elements were scored. Otherwise, the 11 kb sequence centered at the middle of the query exon was scored using SpliceAI when performing exon finding. Cutoffs were chosen to capture a majority of mRNA exons.

### MaxEntScan scoring

We computed MaxEntScan scores using the Python module maxentpy, a Python wrapper for the original MaxEntScan implementation. Cutoffs were chosen to capture a majority of mRNA exons.

### Exon finding on Chromosome 17

We used MaxEntScan and SpliceAI scores to call genome sequences as exons. We computed splice site scores across every base of Chromosome 17 using MaxEntScan and SpliceAI. We used a threshold and checked if the 5′SS score is above this threshold. We then checked if a 3′SS score is also above this threshold within an exon “length” of the selected 3′SS. An exon “length” corresponds to between 63 and 222 bp, the 10% and 90% percentiles of GENCODE mRNA internal exons.

For MaxEntScan we required both splice sites to have a score of at least 6. For SpliceAI we used a threshold of 0.2. We used this threshold because exons with a score delta greater than 0.8 were considered unlikely to splice in the publication associated with SpliceAI. We scored 1000 bp fragments padded with N characters to fit in the 10 kb SpliceAI model.

### Shuffled Chromosome 17

We created a shuffled chromosome preserving dinucleotide frequencies using the fasta-shuffle-letters package from the MEME suite with parameters -kmer 2 -seed 1337. We ran the command on 20 million bp, sequentially, (5 runs) across Chromosome 17 and joined the output into a single chromosome “chr17_shuffle” FASTA file.

### Snaptron exon database

We used the Snaptron SQL database SRAv2 (http://snaptron.cs.jhu.edu/srav2/snaptron?regions=ABCD3) which contains junctions obtained from ∼21,000 samples from the NCBI Sequence Read Archive (SRA; https://www.ncbi.nlm.nih.gov/sra) using the reference hg38.

### HEK293 database

HEK293 gene expression data was obtained from supplemental data for Nieborak et al. (2023) (https://ftp.ncbi.nlm.nih.gov/geo/series/GSE235nnn/GSE235387/suppl/GSE235387_HEK293.xlsx) which contains data for HEK293 wild type control samples. HEK293 transcript RPKM values are from the column “HEK293-WT-S20190326-S20190326”.

### HEK293 percent spliced in

Percent spliced in numbers were obtained from the supplemental data of Ellis et al. (2023) (https://ftp.ncbi.nlm.nih.gov/geo/series/GSE221nnn/GSE221838/suppl/GSE221838_d0_v_d1000_SE.MATS.JCEC.txt.gz).

“IncLevel1”. Splicing inclusion levels containing an “NA” were skipped and PSI values were calculated as the mean of the provided inclusion percents list. Splicing inclusion levels were associated with exon trapping data by finding exons that overlap the splicing inclusion exon list.

### Housekeeping gene list

A list of housekeeping genes was obtained from the supplemental file of Hounkpe et al. (2021), Supplemental Table S1.

### ISS frequency

A list of sequencing displaying ISS activity was obtained from Wen et al. (2010). The list of 5′SS and 3′SS ISS sequences were combined. The frequency of ISS sequences were computed around the 3′SS and 5′SS for the indicated exon data set and the curves were smoothed with a 19 bp moving average. Exons overlapping repeats were not included in the data sets.

### Nonsense mediated decay

Exons used for nonsense mediated decay analysis consist of Internal mRNA exons with length divisible by three and nonzero read counts in the given reading frames. The ratio of NMD is calculated for an exon as follows for two given reading frames: The numerator lacks a stop codon in the first given reading frame vector and the denominator must have one or more stop codons in the second given reading frame vector. Positive values of the log_2_ of this number indicate the fold increase of the exon abundance in the reading frame lacking a stop codon over the reading frame with a stop codon.

## Data access

All raw and processed sequencing data generated in this study have been submitted to the NCBI Gene Expression Omnibus (GEO; https://www.ncbi.nlm.nih.gov/geo/) under accession number GSE213006.

Supplemental data corresponding to figures is available as Supplemental Data and at https://hugheslab.ccbr.utoronto.ca/supplementary-data/ExonTrappingGenome. All code to analyze the data is available as Supplemental Code and at GitHub (https://github.com/nstep2/ExonTrapGenome).

## Supplementary Material

Supplement 1

Supplement 2

Supplement 3

Supplement 4

Supplement 5
