# Supplementary material for: The human genome contains over a million autonomous exons
Source: Genome Res. 2023 Nov;33(11):1865–78. doi: 10.1101/gr.277792.123 (PMC10760453; doi:10.1101/gr.277792.123)
Supplement: Supplement 4 [file Supplemental_figures.pdf]

A)

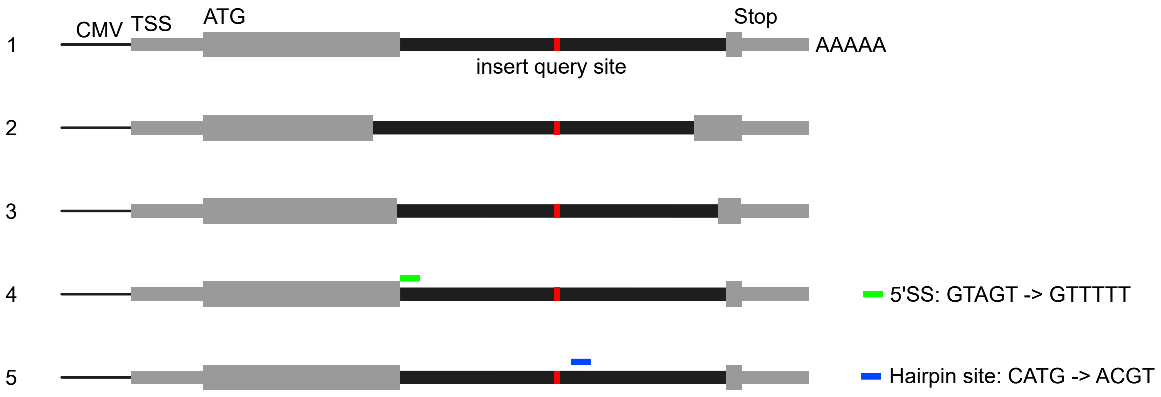

B)

Vector B
Shared exon counts

Vector A
Shared exon counts

	1	2	3	4	5
**1**	892,250	378,742	317,543	325,946	576,377
**2**		609,149	224,971	231,320	404,272
**3**			517,062	194,795	337,672
**4**				516,352	381,412
**5**					931,546

Figure S1

Figure S1. Exon trapping vector design and exon capture.

A) Diagram depicting exon trapping vectors. Vector 1-3 we made by gene synthesis and gateway cloning. Vectors 4 and 5 are derivatives of vector 1, with a 5′SS mutation to weaken the associated MaxEntScan score or a mutation to scramble a putative RBFOX binding site, respectively.

B) Matrix depicting exon detection in each of the vectors and overlap between vectors. The diagonal shows how many of the 1.25 million exons were detected at least once in at least one library generated from each of the vectors. Off-diagonal entries indicate how many exons were detected in both libraries (row and column).

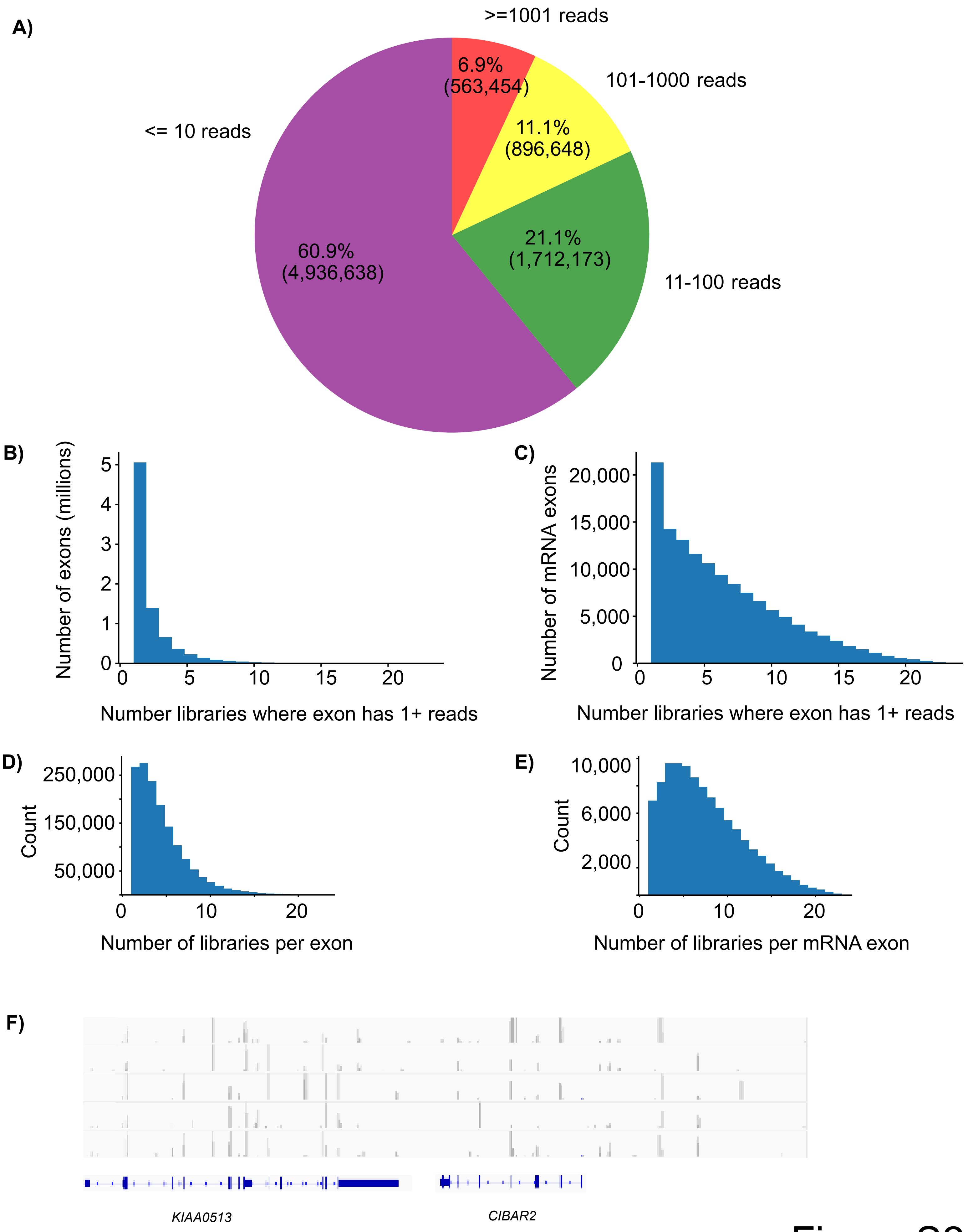
A)
>=1001 reads
101-1000 reads
<= 10 reads
11-100 reads
6.9%
(563,454)
11.1%
(896,648)
21.1%
(1,712,173)
60.9%
(4,936,638)
B)
Number of exons (millions)
Number libraries where exon has 1+ reads
C)
Number of mRNA exons
Number libraries where exon has 1+ reads
D)
Count
Number of libraries per exon
E)
Count
Number of libraries per mRNA exon
F)
KIAA0513
CIBAR2

Figure S2

Figure S2. Exon sequencing reads.

A) Pie chart depicting the fraction and number of sequenced exons at different read count thresholds.
B) Histogram of the number of exons found dependent on the number of sequencing libraries each exon is found within for all exons.
C) Histogram of the number of exons found dependent on the number of sequencing libraries each exon is found within for mRNA exons.
D) Same as B) except for exons with sequencing read counts of 100 or more.
E) Same as C) except for exons with sequencing read counts of 100 or more.
F) Mapped sequencing read counts (combined forward and reverse strand pileups) for the different vector backbones for a region containing *KIAA0513* and a portion of *CIBAR2* (display region coordinates: Chr16:85,062,938-85,134,585). The zoom-in region corresponds to exon 7 of *CIBAR2.*

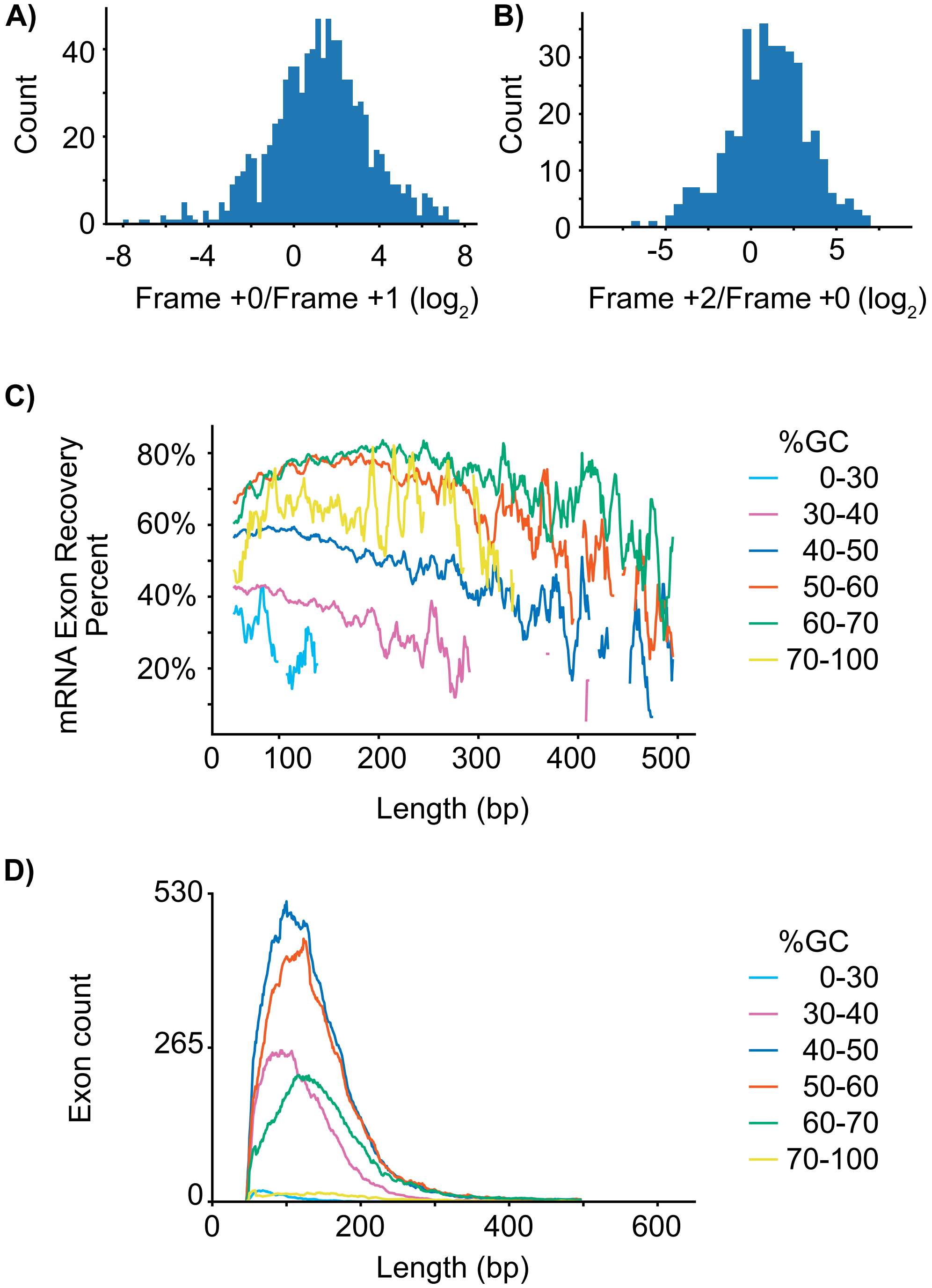
A)
Count
Frame +0/Frame +1 (log2)
B)
Count
Frame +2/Frame +0 (log2)
C)
mRNA Exon Recovery Percent
Length (bp)
%GC
0-30
30-40
40-50
50-60
60-70
70-100
D)
Exon count
Length (bp)
%GC
0-30
30-40
40-50
50-60
60-70
70-100

Figure S3

Figure S3. Exon recovery.

A) Histogram depicting ratio of exon sequencing read counts for different reading frames depending on the presence or absence of stop codons. Exons have no stop codon in frame +0 and at least one stop codon in frame +1.
B) Histogram depicting ratio of exon sequencing read counts for different reading frames depending on the presence or absence of stop codons. Exons have no stop codon in frame +2 and at least one stop codon in frame +0.
C) Line plots depict the distribution mRNA exon recovery percent as a function of exon length, subset by exon GC content. Plots have a 9 bp smoothing window applied.
D) Line plots depict the distribution mRNA exon lengths, subset by exon GC content. Plots have a 9 bp smoothing window applied.

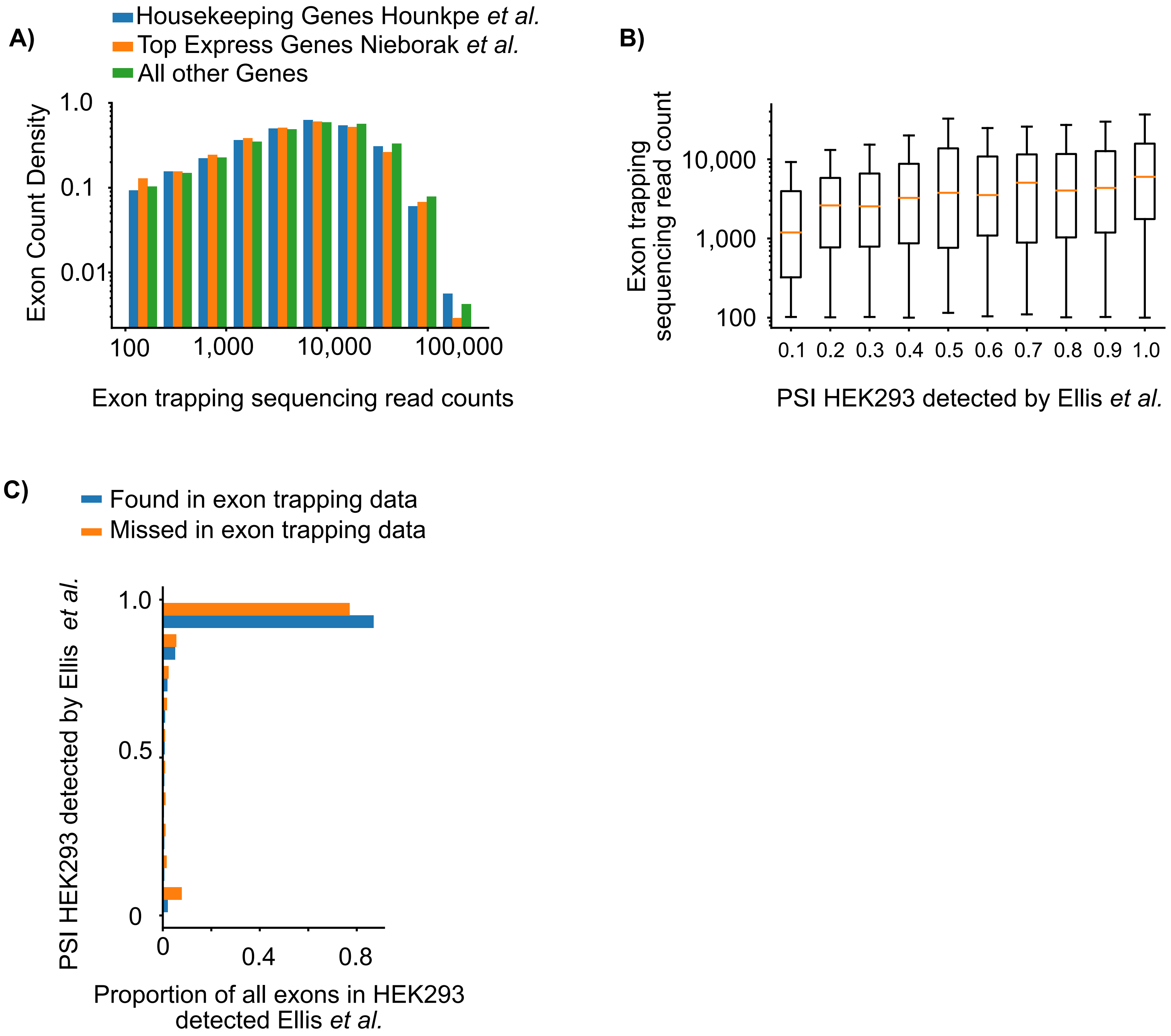
A)
Housekeeping Genes Hounkpe et al.
Top Express Genes Nieborak et al.
All other Genes
Exon Count Density
1.0
0.1
0.01
100
1,000
10,000
100,000
Exon trapping sequencing read counts
B)
Exon trapping sequencing read count
10,000
1,000
100
0.1 0.2 0.3 0.4 0.5 0.6 0.7 0.8 0.9 1.0
PSI HEK293 detected by Ellis et al.
C)
Found in exon trapping data
Missed in exon trapping data
PSI HEK293 detected by Ellis et al.
1.0
0.5
0
0
0.4
0.8
Proportion of all exons in HEK293 detected Ellis et al.

Figure S4

Figure S4. Exon trapping exons HEK293 cell line properties.

A) Bar plots depicting the sequencing read counts of exon trapping exons from three groups of transcribed HEK293 transcripts: housekeeping genes, remaining top 20% HEK293 expression, and remaining bottom 80% expression genes.
B) Boxplot series depicting exon trapping sequencing read count as a function of exon PSI value ranges associated with those exons, for genes expressed in HEK293.
C) Horizontal bar series depicting the proportion of exon belonging to different PSI values in expressed HEK293 genes for two categories of exons: exons found by exon.

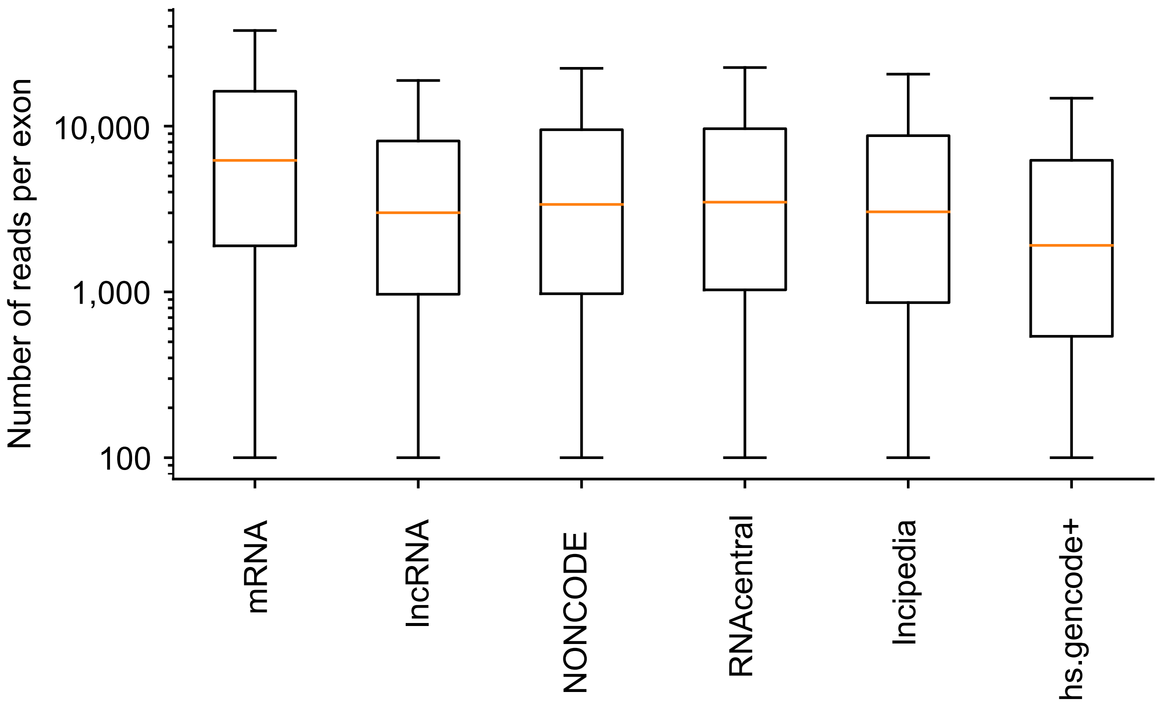
Number of reads per exon
10,000
1,000
100
mRNA
lncRNA
NONCODE
RNAcentral
lncipedia
hs.gencode+

Figure S5

Figure S5. Exon recovery of different lncRNA annotations.

Boxplots showing the number of exon trapping sequencing reads for exons of mRNA, GENCODE lncRNA and exons of different lncRNA annotations with the exons also contained in GENCODE lncRNA removed.

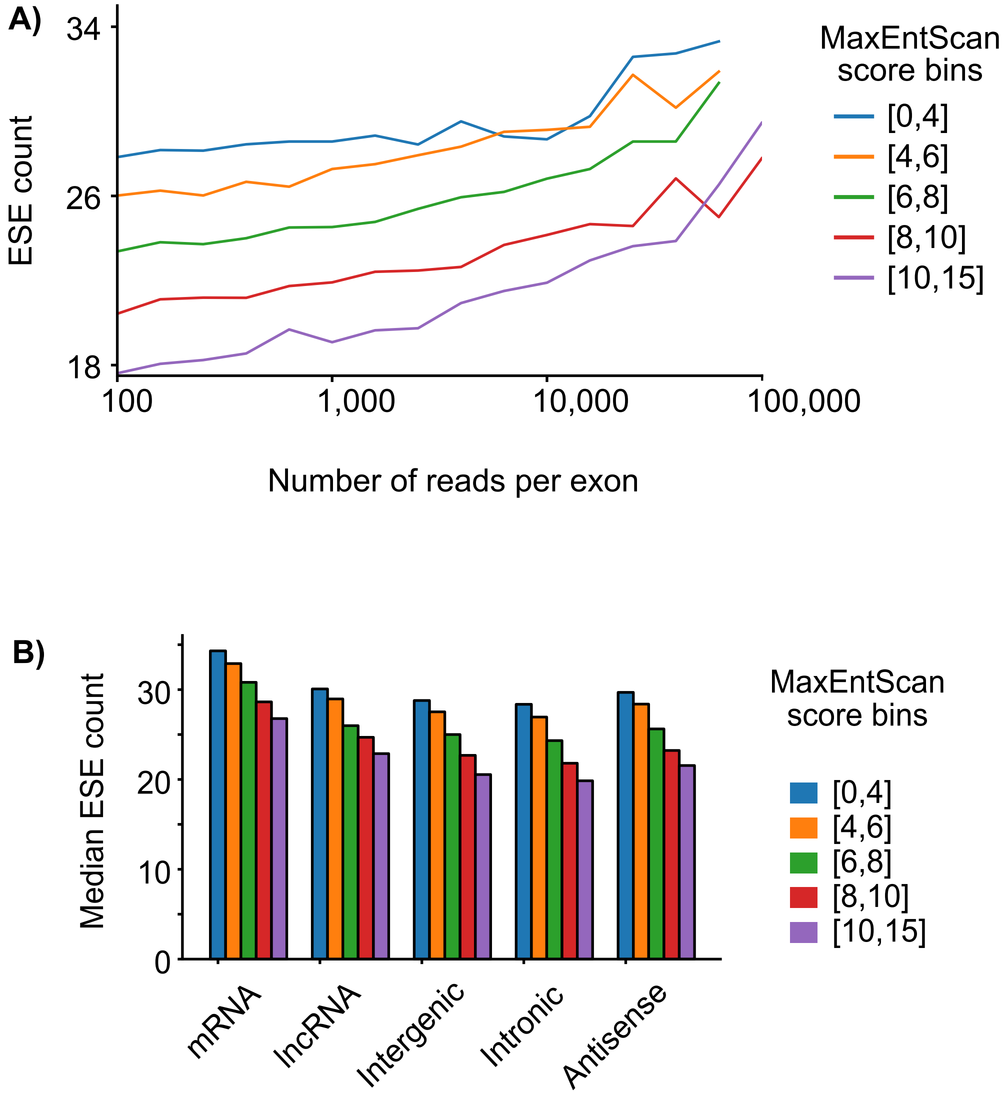
A)
34
26
18
ESE count
100
1,000
10,000
100,000
Number of reads per exon
MaxEntScan score bins
[0,4]
[4,6]
[6,8]
[8,10]
[10,15]
B)
30
20
10
0
Median ESE count
mRNA
lncRNA
Intergenic
Intronic
Antisense
MaxEntScan score bins
[0,4]
[4,6]
[6,8]
[8,10]
[10,15]

Figure S6

Figure S6. Exons splicing enhancer frequencies for different splice site strengths.

A) Line plots showing binned exon read counts verses corresponding median exon ESE counts for intergenic exons, selecting groups defined by both splice site scores being in the specified range. Exons are binned by their sequencing read counts using logarithmic bins ranging from 2 to 5 with 0.2 steps.

B) Bar plots of the median number of splicing enhancers for different exon genomic region categories with exons subdivided into different splice site bins. Exons in splice site bins are required to have both MaxEntScan scores (3′SS and 5′SS) within the indicate range.

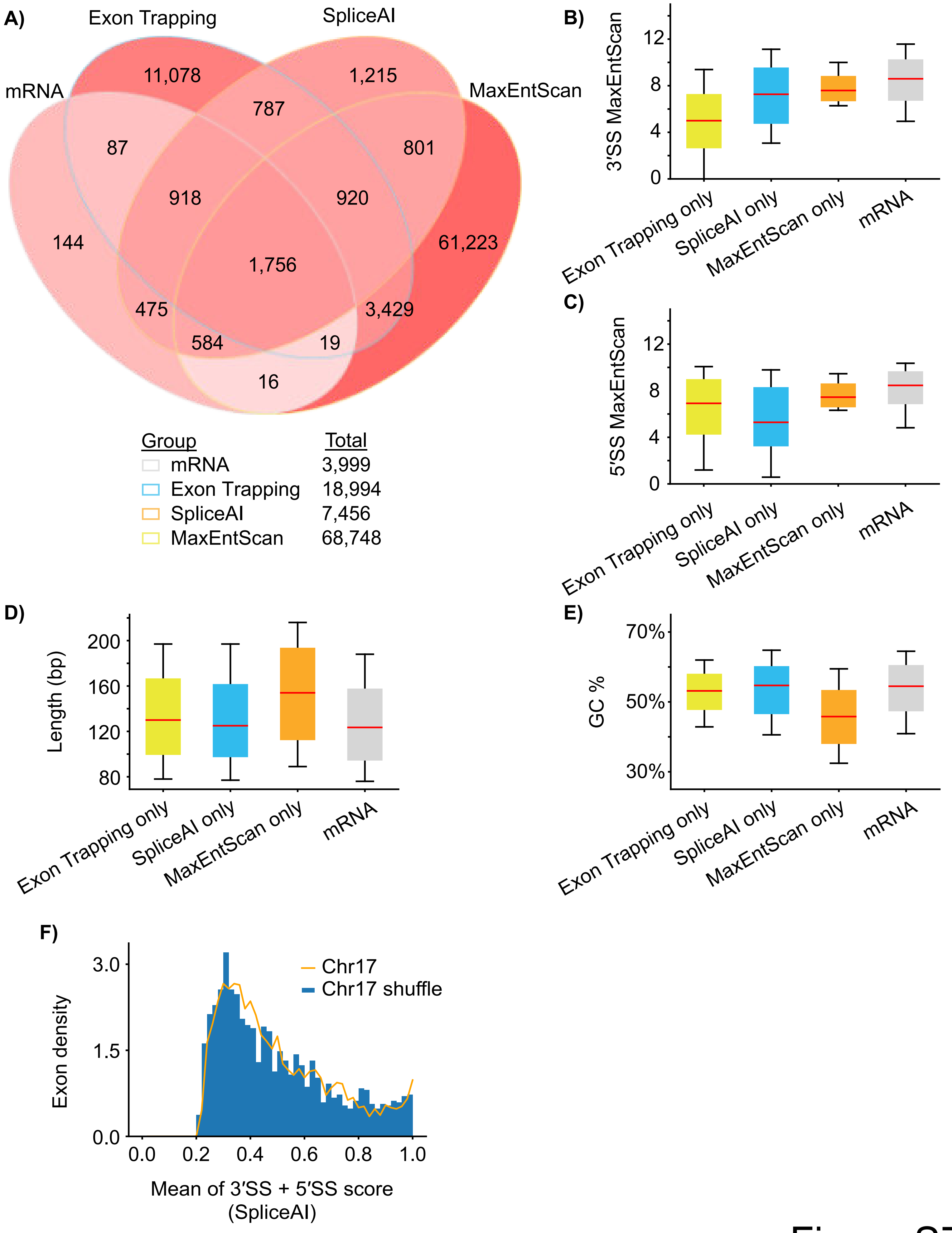
A)
Exon Trapping
SpliceAI
mRNA
MaxEntScan
11,078
1,215
787
87
801
918
920
144
61,223
1,756
475
3,429
584
19
16
Group
Total
mRNA
3,999
Exon Trapping
18,994
SpliceAI
7,456
MaxEntScan
68,748
B)
3′SS MaxEntScan
C)
5′SS MaxEntScan
D)
Length (bp)
E)
GC %
Exon Trapping only
SpliceAI only
MaxEntScan only
mRNA
F)
Exon density
Chr17
Chr17 shuffle
Mean of 3′SS + 5′SS score
(SpliceAI)

Figure S7

Figure S7. Dissection of overlap and properties of exons detected using different approaches.

A) Venn diagram depicting Chromosome 17 forward strand exons found by different exon calling approaches and that share the same 3′SS, but not necessarily 5′SS (annotated mRNA & lncRNA internal exons, exons found by exon trapping, MaxEntScan based exon finding approach, and SpliceAI based exon finding approach). Venn overlaps colored red with intensity determined by (log_{10} #exons)/8.
B) Boxplot of MaxEntScan 3′SS scores for exons belonging only to the indicated exon finding approach.
C) Boxplot of MaxEntScan 5′SS scores for exons belonging only to the indicated exon finding approach.
D) Boxplot of exon lengths for exons belonging only to the indicated exon finding approach.
E) Boxplot of GC content for exons belonging only to the indicated exon finding approach.
F) Histogram showing exon density of mean of 3′SS + corresponding 5′SS SpliceAI score for each unannotated exon found by SpliceAI on Chromosome 17 forward strand (line) and for SpliceAI found exons on a shuffled Chromosome 17 forward strand (bars) for those exons found with SpliceAI score > 0.2. Histograms are area normalized to 1 and depict exon density.

A)

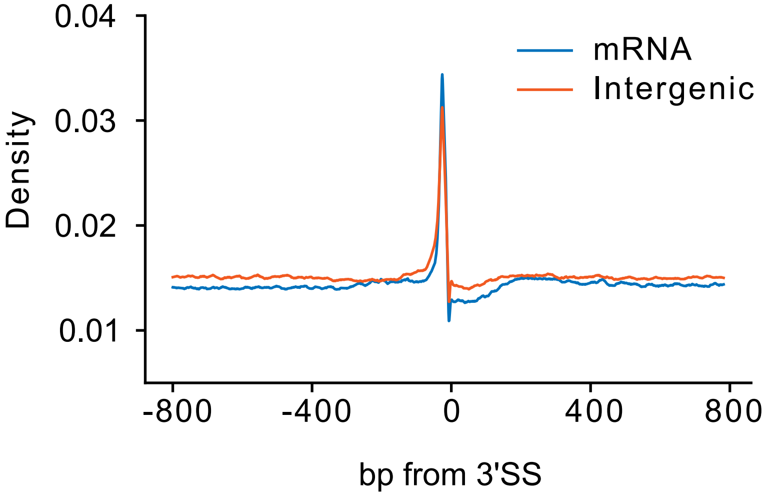

0.04
0.03
0.02
0.01
Density
mRNA
Intergenic
-800
-400
0
400
800
bp from 3'SS
0.04
0.03
0.02
0.01
Density
mRNA
Intergenic
-800
-400
0
400
800
bp from 5'SS

Figure S8

Figure S8. Intronic splice site silencer frequencies around splice sites.

A) Bar graph depicting the frequency of ISS sequences found at a given bp distance from the 3′SS (left) or the 5′SS (right) for mRNA or intergenic exons. Plots are smoothed with a 19 bp moving average. ISS sequences from (Wen et al. 2010).

B) Bar graph depicting the frequency of ISS sequences found at a given bp distance from the 3′SS (left) or the 5′SS (right) for exons unique to different exon finder approaches with the graph of mRNA exon also shown. Plots are smoothed with a 19 bp moving average. ISS sequences from (Wen et al. 2010).
