# Supplementary material for: The human genome contains over a million autonomous exons
Source: Genome Res. 2023 Nov;33(11):1865–78. doi: 10.1101/gr.277792.123 (PMC10760453; doi:10.1101/gr.277792.123)
Supplement: Supplement 5 [file Supplemental_vector_details.docx]

Region highlighting:

CDS

Vector 4 unique change

Vector 5 unique change

Intron reporter: Lower case in between CDS regions

> 1

GACGGATCGGGAGATCTCCCGATCCCCTATGGTGCACTCTCAGTACAATCTGCTCTGATGCCGCATAGTTAAGCCAGTATCTGCTCCCTGCTTGTGTGTTGGAGGTCGCTGAGTAGTGCGCGAGCAAAATTTAAGCTACAACAAGGCAAGGCTTGACCGACAATTGCATGAAGAATCTGCTTAGGGTTAGGCGTTTTGCGCTGCTTCGCGATGTACGGGCCAGATATACGCGTTGACATTGATTATTGACTAGTTATTAATAGTAATCAATTACGGGGTCATTAGTTCATAGCCCATATATGGAGTTCCGCGTTACATAACTTACGGTAAATGGCCCGCCTGGCTGACCGCCCAACGACCCCCGCCCATTGACGTCAATAATGACGTATGTTCCCATAGTAACGCCAATAGGGACTTTCCATTGACGTCAATGGGTGGAGTATTTACGGTAAACTGCCCACTTGGCAGTACATCAAGTGTATCATATGCCAAGTACGCCCCCTATTGACGTCAATGACGGTAAATGGCCCGCCTGGCATTATGCCCAGTACATGACCTTATGGGACTTTCCTACTTGGCAGTACATCTACGTATTAGTCATCGCTATTACCATGGTGATGCGGTTTTGGCAGTACATCAATGGGCGTGGATAGCGGTTTGACTCACGGGGATTTCCAAGTCTCCACCCCATTGACGTCAATGGGAGTTTGTTTTGGCACCAAAATCAACGGGACTTTCCAAAATGTCGTAACAACTCCGCCCCATTGACGCAAATGGGCGGTAGGCGTGTACGGTGGGAGGTCTATATAAGCAGAGCTCTCCCTATCAGTGATAGAGATCTCCCTATCAGTGATAGAGATCGTCGACGAGCTCGTTTAGTGAACCGTCAGATCGCCTGGAGACGCCATCCACGCTGTTTTGACCTCCATAGAAGACACCGGGACCGATCCAGCCTCCGGACTCTAGCGTTTAAACTTAAGCTTggtaccatggtgagcaagggcgaggagctgttcaccggggtggtgcccatcctggtcgagctggacggcgacgtaaacggccacaagttcagcgtgtccggcgagggcgagggcgatgccacctacggcaagctgaccctgaagttcatctgcaccaccggcaagctgcccgtgccctggcccaccctcgtgAccaccctgacctacggcgtgcagtgcttcagccgctaccccgaccacatgaagcagcacgacttcttcaagtccgccatgcccgaaggctacgtccaggagcgcaccatcttcttcAaggacgacggcaactacaagacccgcgccgaggtgaagttcgagggcgacaccctggtgaaccgcatcgagctgaagggcatcgacttcaaggaggacggcaacatcctggggcacaagctggagtacaactacaacagccacaacgtctatatcatggccgacaagcagaagaacggcatcaaggtgaacttcaagatccgccacaacatcgaggacggcagcgtgcagctcgccgaccactaccagcagaacacccccatcggcgacggccccgtgctgctgcccgacaaccactacctgagcacccagtccgccctgagcaaagaccccaacgagaagcgcgatcacatggtcctgctggagttcgtgaccgccgccgggatcactctcggcatggacgagctgtacaagGGGATGGCATCATATCCCTGTCATCAACACGCGAGTGCTTTTGACCAAGCAGCCCGGTCTCGAGGACATAATAACCGACGCACAGCTCTTCGCCCTCGACGGCAACAGGAGGCGACAGAGGTCCGACCGGAGCAAAAGATGCCTACGCTGCTTCGGGTATATATTGACGGCCCGCATGGTATGGGCAAGACGACAACTACGCAACTGCTGGTCGCGTTGGGTAGCCGAGATGACATCGTCTATGTCCCAGAGCCGATGACATACTGGCGGGTGCTCGGAGCAAGCGAGACTATAGCGAACATATACACAACTCAACATCGACTTGATCAAGGCGAAATATCCGCCGGCGATGCGGCGGTGGTGATGACGTCAGCGCAAATTACGATGGGTATGCCCTACGCCGTTACAGATGCGGTTCTTGCTCCTCACATTGGCGGCGAAGCGGGGAGTTCACATGCGCCACCGCCGGCTTTGACGCTTATTTTCGACCGACACCCCATTGCGGCGCTTCTGTGTTACCCGGCGGCAAGGTACCTCATGGGCTCTATGACGCCCCAAGCCGTACTGGCATTTGTCGCCCTTATTCCCCCTACTCTGCCCGGTACGAACATAGTACTTGGAGCGTTGCCTGAAGATAGGCACATTGATCGCCTGGCAAAGAGACAACGGCCCGGCGAACGACTCGATTTGGCTATGCTCGCTGCGATTAGGCGGGTGTACGGGCTGCTGGCGAACACGGTGCGATACCTCCAGTGCGGTGGTAGTTGGAGGGAGGATTGGGGTCAACTCAGCGGCACTGCAGTACCCCCCCAAGGCGCCGAGCCGCAGTCTAATGCCGGCCCACGACCCCACATCGGCGACACACTCTTTACCCTCTTTAGAGCTCCAGAACTGCTTGCTCCCAACGGTGACCTTTACAACGTCTTCGCGTGGGCACTGGACGTCCTGGCGAAGCGACTTCGCTCCATGCACGTGTTCATTCTTGATTACGACCAGTCACCAGCCGGATGCAGAGACGCCCTTTTGCAGTTGACCTCTGGAATGGTTCAGgtaagttatgaggtagaaaggtcaacgtctgaatctcagtacagtgtacagagcagcactatgcttaagttttagcctttgttaaaagctttgctttagtacttttactcgagaaatgcagagtagaaagttaaatcagattctaaggttctgtgtcttaatcaatttaatgtgtaactggatttctgttcaaatattctagaatataaatgggatttaaatgataacttggaatattaataataaaatgtgttttatttttaaacattgaatgaggccaggcgcagtagctcacgcctgtaatcccaacacttcaggaggccaaggcaggcagatcacctgagatccagcctggccaacatggtgaaccccgtctctaccagaaataaaaatttagccggttgtggtggtgcacgcctgtagtcccagctactggcaggtgaattgcttgaacccggcaggcagaggttgcagtgaatttagattgcaccactgcactccactgtaggcaacagagcgagagactcaaaaaaaaaaataaataaatgaatacatagtgaaagcaaaattataggctgaggcaggggaattgcttgagcccgggaatttgagaccagcctgagcagcatagggaacctccacaaaaaatgaggcaggaagccggggaggttgaagctgcagtgagccaagtacgctcccacctgagtctcctcccccagagcaagaccccaccttaaaagaaaaaattatagcctctgtcttagccttttaaaagcaaatttataatggcttataaaagcaaatAAGATAACCGGTTACCAACTGCGGCCGCCtcgacttcataagccattttggaaagccaacacttttcaggcagtttcttgtaatctcttaatgcatgcatcgtacactgagggcaggtaaaatggatgccttgaaagagtccagagaataattttaaagatgttaatttagtatgacaagggtatgtggctttttatttttgccaaaaatttggtatttcggggtttgggtagggaaatgtcaatcatagtttaaaaataaagatatctttgagaatacttccaaagataaaaatgaagtaaaattgtgacattttggtcctcccttgtcaaaatgtgtaatttgacaagtcaaataatgtagttagtaagtattgaccacagttgtcattgataatggtattgaggtatatggatgtctattttgtgcattatttggcaatgtttaagaagttaacagttgtcagtcattgtctgaaagtgagtctgtaagtgtattgttcagcattttatcacccatgaaaaccctttttttatttaaagcatcactctattgaatggtactaaaggtacaaatttgttggggctgagatttttttcttagtttgatttttttttttaagagcatttacttaggaatgttctgtgttctaatactacttatttcagccaccagagagaaccctatgccagtttctaaaaagggtggcaaatcacttgacctgcaagaaacatctcattcatgctctatgaaagtttttattttactaaatgaatgacataattttttaaaacttacaaactgaatgcgtgttgtgttgcctttctgtcttcacagACTCACGTCACGACTCCAGGTTCTATACCAACGATCTGTGATCTCGCTCGAACATTCGCGAGAGAAATGGGTGAGGCCAACTGAGCTCGAGTCTAGAGGGCCCGTTTAAACCCGCTGATCAGCCTCGACTGTGCCTTCTAGTTGCCAGCCATCTGTTGTTTGCCCCTCCCCCGTGCCTTCCTTGACCCTGGAAGGTGCCACTCCCACTGTCCTTTCCTAATAAAATGAGGAAATTGCATCGCATTGTCTGAGTAGGTGTCATTCTATTCTGGGGGGTGGGGTGGGGCAGGACAGCAAGGGGGAGGATTGGGAAGACAATAGCAGGCATGCTGGGGATGCGGTGGGCTCTATGGCTTCTGAGGCGGAAAGAACCAGCTGGGGCTCTAGGGGGTATCCCCACGCGCCCTGTAGCGGCGCATTAAGCGCGGCGGGTGTGGTGGTTACGCGCAGCGTGACCGCTACACTTGCCAGCGCCCTAGCGCCCGCTCCTTTCGCTTTCTTCCCTTCCTTTCTCGCCACGTTCGCCGGCTTTCCCCGTCAAGCTCTAAATCGGGGGCTCCCTTTAGGGTTCCGATTTAGTGCTTTACGGCACCTCGACCCCAAAAAACTTGATTAGGGTGATGGTTCACGTACCTAGAAGTTCCTATTCCGAAGTTCCTATTCTCTAGAAAGTATAGGAACTTCCTTGGCCAAAAAGCCTGAACTCACCGCGACGTCTGTCGAGAAGTTTCTGATCGAAAAGTTCGACAGCGTCTCCGACCTGATGCAGCTCTCGGAGGGCGAAGAATCTCGTGCTTTCAGCTTCGATGTAGGAGGGCGTGGATATGTCCTGCGGGTAAATAGCTGCGCCGATGGTTTCTACAAAGATCGTTATGTTTATCGGCACTTTGCATCGGCCGCGCTCCCGATTCCGGAAGTGCTTGACATTGGGGAgTTCAGCGAGAGCCTGACCTATTGCATCTCCCGCCGTGCACAGGGTGTCACGTTGCAAGACCTGCCTGAAACCGAACTGCCCGCTGTTCTGCAGCCGGTCGCGGAGGCCATGGATGCGATCGCTGCGGCCGATCTTAGCCAGACGAGCGGGTTCGGCCCATTCGGACCGCAAGGAATCGGTCAATACACTACATGGCGTGATTTCATATGCGCGATTGCTGATCCCCATGTGTATCACTGGCAAACTGTGATGGACGACACCGTCAGTGCGTCCGTCGCGCAGGCTCTCGATGAGCTGATGCTTTGGGCCGAGGACTGCCCCGAAGTCCGGCACCTCGTGCACGCGGATTTCGGCTCCAACAATGTCCTGACGGACAATGGCCGCATAACAGCGGTCATTGACTGGAGCGAGGCGATGTTCGGGGATTCCCAATACGAGGTCGCCAACATCTTCTTCTGGAGGCCGTGGTTGGCTTGTATGGAGCAGCAGACGCGCTACTTCGAGCGGAGGCATCCGGAGCTTGCAGGATCGCCGCGGCTCCGGGCGTATATGCTCCGCATTGGTCTTGACCAACTCTATCAGAGCTTGGTTGACGGCAATTTCGATGATGCAGCTTGGGCGCAGGGTCGATGCGACGCAATCGTCCGATCCGGAGCCGGGACTGTCGGGCGTACACAAATCGCCCGCAGAAGCGCGGCCGTCTGGACCGATGGCTGTGTAGAAGTACTCGCCGATAGTGGAAACCGACGCCCCAGCACTCGTCCGAGGGCAAAGGAATAGCACGTACTACGAGATTTCGATTCCACCGCCGCCTTCTATGAAAGGTTGGGCTTCGGAATCGTTTTCCGGGACGCCGGCTGGATGATCCTCCAGCGCGGGGATCTCATGCTGGAGTTCTTCGCCCACCCCAACTTGTTTATTGCAGCTTATAATGGTTACAAATAAAGCAATAGCATCACAAATTTCACAAATAAAGCATTTTTTTCACTGCATTCTAGTTGTGGTTTGTCCAAACTCATCAATGTATCTTATCATGTCTGTATACCGTCGACCTCTAGCTAGAGCTTGGCGTAATCATGGTCATAGCTGTTTCCTGTGTGAAATTGTTATCCGCTCACAATTCCACACAACATACGAGCCGGAAGCATAAAGTGTAAAGCCTGGGGTGCCTAATGAGTGAGCTAACTCACATTAATTGCGTTGCGCTCACTGCCCGCTTTCCAGTCGGGAAACCTGTCGTGCCAGCTGCATTAATGAATCGGCCAACGCGCGGGGAGAGGCGGTTTGCGTATTGGGCGCTCTTCCGCTTCCTCGCTCACTGACTCGCTGCGCTCGGTCGTTCGGCTGCGGCGAGCGGTATCAGCTCACTCAAAGGCGGTAATACGGTTATCCACAGAATCAGGGGATAACGCAGGAAAGAACATGTGAGCAAAAGGCCAGCAAAAGGCCAGGAACCGTAAAAAGGCCGCGTTGCTGGCGTTTTTCCATAGGCTCCGCCCCCCTGACGAGCATCACAAAAATCGACGCTCAAGTCAGAGGTGGCGAAACCCGACAGGACTATAAAGATACCAGGCGTTTCCCCCTGGAAGCTCCCTCGTGCGCTCTCCTGTTCCGACCCTGCCGCTTACCGGATACCTGTCCGCCTTTCTCCCTTCGGGAAGCGTGGCGCTTTCTCATAGCTCACGCTGTAGGTATCTCAGTTCGGTGTAGGTCGTTCGCTCCAAGCTGGGCTGTGTGCACGAACCCCCCGTTCAGCCCGACCGCTGCGCCTTATCCGGTAACTATCGTCTTGAGTCCAACCCGGTAAGACACGACTTATCGCCACTGGCAGCAGCCACTGGTAACAGGATTAGCAGAGCGAGGTATGTAGGCGGTGCTACAGAGTTCTTGAAGTGGTGGCCTAACTACGGCTACACTAGAAGAACAGTATTTGGTATCTGCGCTCTGCTGAAGCCAGTTACCTTCGGAAAAAGAGTTGGTAGCTCTTGATCCGGCAAACAAACCACCGCTGGTAGCGGTGGTTTTTTTGTTTGCAAGCAGCAGATTACGCGCAGAAAAAAAGGATCTCAAGAAGATCCTTTGATCTTTTCTACGGGGTCTGACGCTCAGTGGAACGAAAACTCACGTTAAGGGATTTTGGTCATGAGATTATCAAAAAGGATCTTCACCTAGATCCTTTTAAATTAAAAATGAAGTTTTAAATCAATCTAAAGTATATATGAGTAAACTTGGTCTGACAGTTACCAATGCTTAATCAGTGAGGCACCTATCTCAGCGATCTGTCTATTTCGTTCATCCATAGTTGCCTGACTCCCCGTCGTGTAGATAACTACGATACGGGAGGGCTTACCATCTGGCCCCAGTGCTGCAATGATACCGCGAGACCCACGCTCACCGGCTCCAGATTTATCAGCAATAAACCAGCCAGCCGGAAGGGCCGAGCGCAGAAGTGGTCCTGCAACTTTATCCGCCTCCATCCAGTCTATTAATTGTTGCCGGGAAGCTAGAGTAAGTAGTTCGCCAGTTAATAGTTTGCGCAACGTTGTTGCCATTGCTACAGGCATCGTGGTGTCACGCTCGTCGTTTGGTATGGCTTCATTCAGCTCCGGTTCCCAACGATCAAGGCGAGTTACATGATCCCCCATGTTGTGCAAAAAAGCGGTTAGCTCCTTCGGTCCTCCGATCGTTGTCAGAAGTAAGTTGGCCGCAGTGTTATCACTCATGGTTATGGCAGCACTGCATAATTCTCTTACTGTCATGCCATCCGTAAGATGCTTTTCTGTGACTGGTGAGTACTCAACCAAGTCATTCTGAGAATAGTGTATGCGGCGACCGAGTTGCTCTTGCCCGGCGTCAATACGGGATAATACCGCGCCACATAGCAGAACTTTAAAAGTGCTCATCATTGGAAAACGTTCTTCGGGGCGAAAACTCTCAAGGATCTTACCGCTGTTGAGATCCAGTTCGATGTAACCCACTCGTGCACCCAACTGATCTTCAGCATCTTTTACTTTCACCAGCGTTTCTGGGTGAGCAAAAACAGGAAGGCAAAATGCCGCAAAAAAGGGAATAAGGGCGACACGGAAATGTTGAATACTCATACTCTTCCTTTTTCAATATTATTGAAGCATTTATCAGGGTTATTGTCTCATGAGCGGATACATATTTGAATGTATTTAGAAAAATAAACAAATAGGGGTTCCGCGCACATTTCCCCGAAAAGTGCCACCTGACGTC

> 2

GACGGATCGGGAGATCTCCCGATCCCCTATGGTGCACTCTCAGTACAATCTGCTCTGATGCCGCATAGTTAAGCCAGTATCTGCTCCCTGCTTGTGTGTTGGAGGTCGCTGAGTAGTGCGCGAGCAAAATTTAAGCTACAACAAGGCAAGGCTTGACCGACAATTGCATGAAGAATCTGCTTAGGGTTAGGCGTTTTGCGCTGCTTCGCGATGTACGGGCCAGATATACGCGTTGACATTGATTATTGACTAGTTATTAATAGTAATCAATTACGGGGTCATTAGTTCATAGCCCATATATGGAGTTCCGCGTTACATAACTTACGGTAAATGGCCCGCCTGGCTGACCGCCCAACGACCCCCGCCCATTGACGTCAATAATGACGTATGTTCCCATAGTAACGCCAATAGGGACTTTCCATTGACGTCAATGGGTGGAGTATTTACGGTAAACTGCCCACTTGGCAGTACATCAAGTGTATCATATGCCAAGTACGCCCCCTATTGACGTCAATGACGGTAAATGGCCCGCCTGGCATTATGCCCAGTACATGACCTTATGGGACTTTCCTACTTGGCAGTACATCTACGTATTAGTCATCGCTATTACCATGGTGATGCGGTTTTGGCAGTACATCAATGGGCGTGGATAGCGGTTTGACTCACGGGGATTTCCAAGTCTCCACCCCATTGACGTCAATGGGAGTTTGTTTTGGCACCAAAATCAACGGGACTTTCCAAAATGTCGTAACAACTCCGCCCCATTGACGCAAATGGGCGGTAGGCGTGTACGGTGGGAGGTCTATATAAGCAGAGCTCTCCCTATCAGTGATAGAGATCTCCCTATCAGTGATAGAGATCGTCGACGAGCTCGTTTAGTGAACCGTCAGATCGCCTGGAGACGCCATCCACGCTGTTTTGACCTCCATAGAAGACACCGGGACCGATCCAGCCTCCGGACTCTAGCGTTTAAACTTAAGCTTggtaccatggtgagcaagggcgaggagctgttcaccggggtggtgcccatcctggtcgagctggacggcgacgtaaacggccacaagttcagcgtgtccggcgagggcgagggcgatgccacctacggcaagctgaccctgaagttcatctgcaccaccggcaagctgcccgtgccctggcccaccctcgtgAccaccctgacctacggcgtgcagtgcttcagccgctaccccgaccacatgaagcagcacgacttcttcaagtccgccatgcccgaaggctacgtccaggagcgcaccatcttcttcAaggacgacggcaactacaagacccgcgccgaggtgaagttcgagggcgacaccctggtgaaccgcatcgagctgaagggcatcgacttcaaggaggacggcaacatcctggggcacaagctggagtacaactacaacagccacaacgtctatatcatggccgacaagcagaagaacggcatcaaggtgaacttcaagatccgccacaacatcgaggacggcagcgtgcagctcgccgaccactaccagcagaacacccccatcggcgacggccccgtgctgctgcccgacaaccactacctgagcacccagtccgccctgagcaaagaccccaacgagaagcgcgatcacatggtcctgctggagttcgtgaccgccgccgggatcactctcggcatggacgagctgtacaagGGGATGGCATCATATCCCTGTCATCAACACGCGAGTGCTTTTGACCAAGCAGCCCGGTCTCGAGGACATAATAACCGACGCACAGCTCTTCGCCCTCGACGGCAACAGGAGGCGACAGAGGTCCGACCGGAGCAAAAGATGCCTACGCTGCTTCGGGTATATATTGACGGCCCGCATGGTATGGGCAAGACGACAACTACGCAACTGCTGGTCGCGTTGGGTAGCCGAGATGACATCGTCTATGTCCCAGAGCCGATGACATACTGGCGGGTGCTCGGAGCAAGCGAGACTATAGCGAACATATACACAACTCAACATCGACTTGATCAAGGCGAAATATCCGCCGGCGATGCGGCGGTGGTGATGACGTCAGCGCAAATTACGATGGGTATGCCCTACGCCGTTACAGATGCGGTTCTTGCTCCTCACATTGGCGGCGAAGCGGGGAGTTCACATGCGCCACCGCCGGCTTTGACGCTTATTTTCGACCGACACCCCATTGCGGCGCTTCTGTGTTACCCGGCGGCAAGGTACCTCATGGGCTCTATGACGCCCCAAGCCGTACTGGCATTTGTCGCCCTTATTCCCCCTACTCTGCCCGGTACGAACATAGTACTTGGAGCGTTGCCTGAAGATAGGCACATTGATCGCCTGGCAAAGAGACAACGGCCCGGCGAACGACTCGATTTGGCTATGCTCGCTGCGATTAGGCGGGTGTACGGGCTGCTGGCGAACACGGTGCGATACCTCCAGTGCGGTGGTAGTTGGAGGGAGGATTGGGGTCAACTCAGCGGCACTGCAGTACCCCCCCAAGGCGCCGAGCCGCAGTCTAATGCCGGCCCACGACCCCACATCGGCGACACACTCTTTACCCTCTTTAGAGCTCCAGAACTGCTTGCTCCCAACGGTGACCTTTACAACGTCTTCGCGTGGGCACTGGACGTCCTGGCGAAGCGACTTCGCTCCATGCACGTGTTCATTCTTGATTACGACCAGTCACCAGCCGGATGCAGgtaagttatgaggtagaaaggtcaacgtctgaatctcagtacagtgtacagagcagcactatgcttaagttttagcctttgttaaaagctttgctttagtacttttactcgagaaatgcagagtagaaagttaaatcagattctaaggttctgtgtcttaatcaatttaatgtgtaactggatttctgttcaaatattctagaatataaatgggatttaaatgataacttggaatattaataataaaatgtgttttatttttaaacattgaatgaggccaggcgcagtagctcacgcctgtaatcccaacacttcaggaggccaaggcaggcagatcacctgagatccagcctggccaacatggtgaaccccgtctctaccagaaataaaaatttagccggttgtggtggtgcacgcctgtagtcccagctactggcaggtgaattgcttgaacccggcaggcagaggttgcagtgaatttagattgcaccactgcactccactgtaggcaacagagcgagagactcaaaaaaaaaaataaataaatgaatacatagtgaaagcaaaattataggctgaggcaggggaattgcttgagcccgggaatttgagaccagcctgagcagcatagggaacctccacaaaaaatgaggcaggaagccggggaggttgaagctgcagtgagccaagtacgctcccacctgagtctcctcccccagagcaagaccccaccttaaaagaaaaaattatagcctctgtcttagccttttaaaagcaaatttataatggcttataaaagcaaatAAGATAACCGGTTACCAACTGCGGCCGCCtcgacttcataagccattttggaaagccaacacttttcaggcagtttcttgtaatctcttaatgcatgcatcgtacactgagggcaggtaaaatggatgccttgaaagagtccagagaataattttaaagatgttaatttagtatgacaagggtatgtggctttttatttttgccaaaaatttggtatttcggggtttgggtagggaaatgtcaatcatagtttaaaaataaagatatctttgagaatacttccaaagataaaaatgaagtaaaattgtgacattttggtcctcccttgtcaaaatgtgtaatttgacaagtcaaataatgtagttagtaagtattgaccacagttgtcattgataatggtattgaggtatatggatgtctattttgtgcattatttggcaatgtttaagaagttaacagttgtcagtcattgtctgaaagtgagtctgtaagtgtattgttcagcattttatcacccatgaaaaccctttttttatttaaagcatcactctattgaatggtactaaaggtacaaatttgttggggctgagatttttttcttagtttgatttttttttttaagagcatttacttaggaatgttctgtgttctaatactacttatttcagccaccagagagaaccctatgccagtttctaaaaagggtggcaaatcacttgacctgcaagaaacatctcattcatgctctatgaaagtttttattttactaaatgaatgacataattttttaaaacttacaaactgaatgcgtgttgtgttgcctttctgtcttcacagAGACGCCCTTTTGCAGTTGACCTCTGGAATGGTTCAGACTCACGTCACGACTCCAGGTTCTATACCAACGATCTGTGATCTCGCTCGAACATTCGCGAGAGAAATGGGTGAGGCCAACTGAGCTCGAGTCTAGAGGGCCCGTTTAAACCCGCTGATCAGCCTCGACTGTGCCTTCTAGTTGCCAGCCATCTGTTGTTTGCCCCTCCCCCGTGCCTTCCTTGACCCTGGAAGGTGCCACTCCCACTGTCCTTTCCTAATAAAATGAGGAAATTGCATCGCATTGTCTGAGTAGGTGTCATTCTATTCTGGGGGGTGGGGTGGGGCAGGACAGCAAGGGGGAGGATTGGGAAGACAATAGCAGGCATGCTGGGGATGCGGTGGGCTCTATGGCTTCTGAGGCGGAAAGAACCAGCTGGGGCTCTAGGGGGTATCCCCACGCGCCCTGTAGCGGCGCATTAAGCGCGGCGGGTGTGGTGGTTACGCGCAGCGTGACCGCTACACTTGCCAGCGCCCTAGCGCCCGCTCCTTTCGCTTTCTTCCCTTCCTTTCTCGCCACGTTCGCCGGCTTTCCCCGTCAAGCTCTAAATCGGGGGCTCCCTTTAGGGTTCCGATTTAGTGCTTTACGGCACCTCGACCCCAAAAAACTTGATTAGGGTGATGGTTCACGTACCTAGAAGTTCCTATTCCGAAGTTCCTATTCTCTAGAAAGTATAGGAACTTCCTTGGCCAAAAAGCCTGAACTCACCGCGACGTCTGTCGAGAAGTTTCTGATCGAAAAGTTCGACAGCGTCTCCGACCTGATGCAGCTCTCGGAGGGCGAAGAATCTCGTGCTTTCAGCTTCGATGTAGGAGGGCGTGGATATGTCCTGCGGGTAAATAGCTGCGCCGATGGTTTCTACAAAGATCGTTATGTTTATCGGCACTTTGCATCGGCCGCGCTCCCGATTCCGGAAGTGCTTGACATTGGGGAgTTCAGCGAGAGCCTGACCTATTGCATCTCCCGCCGTGCACAGGGTGTCACGTTGCAAGACCTGCCTGAAACCGAACTGCCCGCTGTTCTGCAGCCGGTCGCGGAGGCCATGGATGCGATCGCTGCGGCCGATCTTAGCCAGACGAGCGGGTTCGGCCCATTCGGACCGCAAGGAATCGGTCAATACACTACATGGCGTGATTTCATATGCGCGATTGCTGATCCCCATGTGTATCACTGGCAAACTGTGATGGACGACACCGTCAGTGCGTCCGTCGCGCAGGCTCTCGATGAGCTGATGCTTTGGGCCGAGGACTGCCCCGAAGTCCGGCACCTCGTGCACGCGGATTTCGGCTCCAACAATGTCCTGACGGACAATGGCCGCATAACAGCGGTCATTGACTGGAGCGAGGCGATGTTCGGGGATTCCCAATACGAGGTCGCCAACATCTTCTTCTGGAGGCCGTGGTTGGCTTGTATGGAGCAGCAGACGCGCTACTTCGAGCGGAGGCATCCGGAGCTTGCAGGATCGCCGCGGCTCCGGGCGTATATGCTCCGCATTGGTCTTGACCAACTCTATCAGAGCTTGGTTGACGGCAATTTCGATGATGCAGCTTGGGCGCAGGGTCGATGCGACGCAATCGTCCGATCCGGAGCCGGGACTGTCGGGCGTACACAAATCGCCCGCAGAAGCGCGGCCGTCTGGACCGATGGCTGTGTAGAAGTACTCGCCGATAGTGGAAACCGACGCCCCAGCACTCGTCCGAGGGCAAAGGAATAGCACGTACTACGAGATTTCGATTCCACCGCCGCCTTCTATGAAAGGTTGGGCTTCGGAATCGTTTTCCGGGACGCCGGCTGGATGATCCTCCAGCGCGGGGATCTCATGCTGGAGTTCTTCGCCCACCCCAACTTGTTTATTGCAGCTTATAATGGTTACAAATAAAGCAATAGCATCACAAATTTCACAAATAAAGCATTTTTTTCACTGCATTCTAGTTGTGGTTTGTCCAAACTCATCAATGTATCTTATCATGTCTGTATACCGTCGACCTCTAGCTAGAGCTTGGCGTAATCATGGTCATAGCTGTTTCCTGTGTGAAATTGTTATCCGCTCACAATTCCACACAACATACGAGCCGGAAGCATAAAGTGTAAAGCCTGGGGTGCCTAATGAGTGAGCTAACTCACATTAATTGCGTTGCGCTCACTGCCCGCTTTCCAGTCGGGAAACCTGTCGTGCCAGCTGCATTAATGAATCGGCCAACGCGCGGGGAGAGGCGGTTTGCGTATTGGGCGCTCTTCCGCTTCCTCGCTCACTGACTCGCTGCGCTCGGTCGTTCGGCTGCGGCGAGCGGTATCAGCTCACTCAAAGGCGGTAATACGGTTATCCACAGAATCAGGGGATAACGCAGGAAAGAACATGTGAGCAAAAGGCCAGCAAAAGGCCAGGAACCGTAAAAAGGCCGCGTTGCTGGCGTTTTTCCATAGGCTCCGCCCCCCTGACGAGCATCACAAAAATCGACGCTCAAGTCAGAGGTGGCGAAACCCGACAGGACTATAAAGATACCAGGCGTTTCCCCCTGGAAGCTCCCTCGTGCGCTCTCCTGTTCCGACCCTGCCGCTTACCGGATACCTGTCCGCCTTTCTCCCTTCGGGAAGCGTGGCGCTTTCTCATAGCTCACGCTGTAGGTATCTCAGTTCGGTGTAGGTCGTTCGCTCCAAGCTGGGCTGTGTGCACGAACCCCCCGTTCAGCCCGACCGCTGCGCCTTATCCGGTAACTATCGTCTTGAGTCCAACCCGGTAAGACACGACTTATCGCCACTGGCAGCAGCCACTGGTAACAGGATTAGCAGAGCGAGGTATGTAGGCGGTGCTACAGAGTTCTTGAAGTGGTGGCCTAACTACGGCTACACTAGAAGAACAGTATTTGGTATCTGCGCTCTGCTGAAGCCAGTTACCTTCGGAAAAAGAGTTGGTAGCTCTTGATCCGGCAAACAAACCACCGCTGGTAGCGGTGGTTTTTTTGTTTGCAAGCAGCAGATTACGCGCAGAAAAAAAGGATCTCAAGAAGATCCTTTGATCTTTTCTACGGGGTCTGACGCTCAGTGGAACGAAAACTCACGTTAAGGGATTTTGGTCATGAGATTATCAAAAAGGATCTTCACCTAGATCCTTTTAAATTAAAAATGAAGTTTTAAATCAATCTAAAGTATATATGAGTAAACTTGGTCTGACAGTTACCAATGCTTAATCAGTGAGGCACCTATCTCAGCGATCTGTCTATTTCGTTCATCCATAGTTGCCTGACTCCCCGTCGTGTAGATAACTACGATACGGGAGGGCTTACCATCTGGCCCCAGTGCTGCAATGATACCGCGAGACCCACGCTCACCGGCTCCAGATTTATCAGCAATAAACCAGCCAGCCGGAAGGGCCGAGCGCAGAAGTGGTCCTGCAACTTTATCCGCCTCCATCCAGTCTATTAATTGTTGCCGGGAAGCTAGAGTAAGTAGTTCGCCAGTTAATAGTTTGCGCAACGTTGTTGCCATTGCTACAGGCATCGTGGTGTCACGCTCGTCGTTTGGTATGGCTTCATTCAGCTCCGGTTCCCAACGATCAAGGCGAGTTACATGATCCCCCATGTTGTGCAAAAAAGCGGTTAGCTCCTTCGGTCCTCCGATCGTTGTCAGAAGTAAGTTGGCCGCAGTGTTATCACTCATGGTTATGGCAGCACTGCATAATTCTCTTACTGTCATGCCATCCGTAAGATGCTTTTCTGTGACTGGTGAGTACTCAACCAAGTCATTCTGAGAATAGTGTATGCGGCGACCGAGTTGCTCTTGCCCGGCGTCAATACGGGATAATACCGCGCCACATAGCAGAACTTTAAAAGTGCTCATCATTGGAAAACGTTCTTCGGGGCGAAAACTCTCAAGGATCTTACCGCTGTTGAGATCCAGTTCGATGTAACCCACTCGTGCACCCAACTGATCTTCAGCATCTTTTACTTTCACCAGCGTTTCTGGGTGAGCAAAAACAGGAAGGCAAAATGCCGCAAAAAAGGGAATAAGGGCGACACGGAAATGTTGAATACTCATACTCTTCCTTTTTCAATATTATTGAAGCATTTATCAGGGTTATTGTCTCATGAGCGGATACATATTTGAATGTATTTAGAAAAATAAACAAATAGGGGTTCCGCGCACATTTCCCCGAAAAGTGCCACCTGACGTC

> 3

GACGGATCGGGAGATCTCCCGATCCCCTATGGTGCACTCTCAGTACAATCTGCTCTGATGCCGCATAGTTAAGCCAGTATCTGCTCCCTGCTTGTGTGTTGGAGGTCGCTGAGTAGTGCGCGAGCAAAATTTAAGCTACAACAAGGCAAGGCTTGACCGACAATTGCATGAAGAATCTGCTTAGGGTTAGGCGTTTTGCGCTGCTTCGCGATGTACGGGCCAGATATACGCGTTGACATTGATTATTGACTAGTTATTAATAGTAATCAATTACGGGGTCATTAGTTCATAGCCCATATATGGAGTTCCGCGTTACATAACTTACGGTAAATGGCCCGCCTGGCTGACCGCCCAACGACCCCCGCCCATTGACGTCAATAATGACGTATGTTCCCATAGTAACGCCAATAGGGACTTTCCATTGACGTCAATGGGTGGAGTATTTACGGTAAACTGCCCACTTGGCAGTACATCAAGTGTATCATATGCCAAGTACGCCCCCTATTGACGTCAATGACGGTAAATGGCCCGCCTGGCATTATGCCCAGTACATGACCTTATGGGACTTTCCTACTTGGCAGTACATCTACGTATTAGTCATCGCTATTACCATGGTGATGCGGTTTTGGCAGTACATCAATGGGCGTGGATAGCGGTTTGACTCACGGGGATTTCCAAGTCTCCACCCCATTGACGTCAATGGGAGTTTGTTTTGGCACCAAAATCAACGGGACTTTCCAAAATGTCGTAACAACTCCGCCCCATTGACGCAAATGGGCGGTAGGCGTGTACGGTGGGAGGTCTATATAAGCAGAGCTCTCCCTATCAGTGATAGAGATCTCCCTATCAGTGATAGAGATCGTCGACGAGCTCGTTTAGTGAACCGTCAGATCGCCTGGAGACGCCATCCACGCTGTTTTGACCTCCATAGAAGACACCGGGACCGATCCAGCCTCCGGACTCTAGCGTTTAAACTTAAGCTTggtaccatggtgagcaagggcgaggagctgttcaccggggtggtgcccatcctggtcgagctggacggcgacgtaaacggccacaagttcagcgtgtccggcgagggcgagggcgatgccacctacggcaagctgaccctgaagttcatctgcaccaccggcaagctgcccgtgccctggcccaccctcgtgAccaccctgacctacggcgtgcagtgcttcagccgctaccccgaccacatgaagcagcacgacttcttcaagtccgccatgcccgaaggctacgtccaggagcgcaccatcttcttcAaggacgacggcaactacaagacccgcgccgaggtgaagttcgagggcgacaccctggtgaaccgcatcgagctgaagggcatcgacttcaaggaggacggcaacatcctggggcacaagctggagtacaactacaacagccacaacgtctatatcatggccgacaagcagaagaacggcatcaaggtgaacttcaagatccgccacaacatcgaggacggcagcgtgcagctcgccgaccactaccagcagaacacccccatcggcgacggccccgtgctgctgcccgacaaccactacctgagcacccagtccgccctgagcaaagaccccaacgagaagcgcgatcacatggtcctgctggagttcgtgaccgccgccgggatcactctcggcatggacgagctgtacaagGGGATGGCATCATATCCCTGTCATCAACACGCGAGTGCTTTTGACCAAGCAGCCCGGTCTCGAGGACATAATAACCGACGCACAGCTCTTCGCCCTCGACGGCAACAGGAGGCGACAGAGGTCCGACCGGAGCAAAAGATGCCTACGCTGCTTCGGGTATATATTGACGGCCCGCATGGTATGGGCAAGACGACAACTACGCAACTGCTGGTCGCGTTGGGTAGCCGAGATGACATCGTCTATGTCCCAGAGCCGATGACATACTGGCGGGTGCTCGGAGCAAGCGAGACTATAGCGAACATATACACAACTCAACATCGACTTGATCAAGGCGAAATATCCGCCGGCGATGCGGCGGTGGTGATGACGTCAGCGCAAATTACGATGGGTATGCCCTACGCCGTTACAGATGCGGTTCTTGCTCCTCACATTGGCGGCGAAGCGGGGAGTTCACATGCGCCACCGCCGGCTTTGACGCTTATTTTCGACCGACACCCCATTGCGGCGCTTCTGTGTTACCCGGCGGCAAGGTACCTCATGGGCTCTATGACGCCCCAAGCCGTACTGGCATTTGTCGCCCTTATTCCCCCTACTCTGCCCGGTACGAACATAGTACTTGGAGCGTTGCCTGAAGATAGGCACATTGATCGCCTGGCAAAGAGACAACGGCCCGGCGAACGACTCGATTTGGCTATGCTCGCTGCGATTAGGCGGGTGTACGGGCTGCTGGCGAACACGGTGCGATACCTCCAGTGCGGTGGTAGTTGGAGGGAGGATTGGGGTCAACTCAGCGGCACTGCAGTACCCCCCCAAGGCGCCGAGCCGCAGTCTAATGCCGGCCCACGACCCCACATCGGCGACACACTCTTTACCCTCTTTAGAGCTCCAGgtaagttatgaggtagaaaggtcaacgtctgaatctcagtacagtgtacagagcagcactatgcttaagttttagcctttgttaaaagctttgctttagtacttttactcgagaaatgcagagtagaaagttaaatcagattctaaggttctgtgtcttaatcaatttaatgtgtaactggatttctgttcaaatattctagaatataaatgggatttaaatgataacttggaatattaataataaaatgtgttttatttttaaacattgaatgaggccaggcgcagtagctcacgcctgtaatcccaacacttcaggaggccaaggcaggcagatcacctgagatccagcctggccaacatggtgaaccccgtctctaccagaaataaaaatttagccggttgtggtggtgcacgcctgtagtcccagctactggcaggtgaattgcttgaacccggcaggcagaggttgcagtgaatttagattgcaccactgcactccactgtaggcaacagagcgagagactcaaaaaaaaaaataaataaatgaatacatagtgaaagcaaaattataggctgaggcaggggaattgcttgagcccgggaatttgagaccagcctgagcagcatagggaacctccacaaaaaatgaggcaggaagccggggaggttgaagctgcagtgagccaagtacgctcccacctgagtctcctcccccagagcaagaccccaccttaaaagaaaaaattatagcctctgtcttagccttttaaaagcaaatttataatggcttataaaagcaaatAAGATAACCGGTTACCAACTGCGGCCGCCtcgacttcataagccattttggaaagccaacacttttcaggcagtttcttgtaatctcttaatgcatgcatcgtacactgagggcaggtaaaatggatgccttgaaagagtccagagaataattttaaagatgttaatttagtatgacaagggtatgtggctttttatttttgccaaaaatttggtatttcggggtttgggtagggaaatgtcaatcatagtttaaaaataaagatatctttgagaatacttccaaagataaaaatgaagtaaaattgtgacattttggtcctcccttgtcaaaatgtgtaatttgacaagtcaaataatgtagttagtaagtattgaccacagttgtcattgataatggtattgaggtatatggatgtctattttgtgcattatttggcaatgtttaagaagttaacagttgtcagtcattgtctgaaagtgagtctgtaagtgtattgttcagcattttatcacccatgaaaaccctttttttatttaaagcatcactctattgaatggtactaaaggtacaaatttgttggggctgagatttttttcttagtttgatttttttttttaagagcatttacttaggaatgttctgtgttctaatactacttatttcagccaccagagagaaccctatgccagtttctaaaaagggtggcaaatcacttgacctgcaagaaacatctcattcatgctctatgaaagtttttattttactaaatgaatgacataattttttaaaacttacaaactgaatgcgtgttgtgttgcctttctgtcttcacagAACTGCTTGCTCCCAACGGTGACCTTTACAACGTCTTCGCGTGGGCACTGGACGTCCTGGCGAAGCGACTTCGCTCCATGCACGTGTTCATTCTTGATTACGACCAGTCACCAGCCGGATGCAGAGACGCCCTTTTGCAGTTGACCTCTGGAATGGTTCAGACTCACGTCACGACTCCAGGTTCTATACCAACGATCTGTGATCTCGCTCGAACATTCGCGAGAGAAATGGGTGAGGCCAACTGAGCTCGAGTCTAGAGGGCCCGTTTAAACCCGCTGATCAGCCTCGACTGTGCCTTCTAGTTGCCAGCCATCTGTTGTTTGCCCCTCCCCCGTGCCTTCCTTGACCCTGGAAGGTGCCACTCCCACTGTCCTTTCCTAATAAAATGAGGAAATTGCATCGCATTGTCTGAGTAGGTGTCATTCTATTCTGGGGGGTGGGGTGGGGCAGGACAGCAAGGGGGAGGATTGGGAAGACAATAGCAGGCATGCTGGGGATGCGGTGGGCTCTATGGCTTCTGAGGCGGAAAGAACCAGCTGGGGCTCTAGGGGGTATCCCCACGCGCCCTGTAGCGGCGCATTAAGCGCGGCGGGTGTGGTGGTTACGCGCAGCGTGACCGCTACACTTGCCAGCGCCCTAGCGCCCGCTCCTTTCGCTTTCTTCCCTTCCTTTCTCGCCACGTTCGCCGGCTTTCCCCGTCAAGCTCTAAATCGGGGGCTCCCTTTAGGGTTCCGATTTAGTGCTTTACGGCACCTCGACCCCAAAAAACTTGATTAGGGTGATGGTTCACGTACCTAGAAGTTCCTATTCCGAAGTTCCTATTCTCTAGAAAGTATAGGAACTTCCTTGGCCAAAAAGCCTGAACTCACCGCGACGTCTGTCGAGAAGTTTCTGATCGAAAAGTTCGACAGCGTCTCCGACCTGATGCAGCTCTCGGAGGGCGAAGAATCTCGTGCTTTCAGCTTCGATGTAGGAGGGCGTGGATATGTCCTGCGGGTAAATAGCTGCGCCGATGGTTTCTACAAAGATCGTTATGTTTATCGGCACTTTGCATCGGCCGCGCTCCCGATTCCGGAAGTGCTTGACATTGGGGAgTTCAGCGAGAGCCTGACCTATTGCATCTCCCGCCGTGCACAGGGTGTCACGTTGCAAGACCTGCCTGAAACCGAACTGCCCGCTGTTCTGCAGCCGGTCGCGGAGGCCATGGATGCGATCGCTGCGGCCGATCTTAGCCAGACGAGCGGGTTCGGCCCATTCGGACCGCAAGGAATCGGTCAATACACTACATGGCGTGATTTCATATGCGCGATTGCTGATCCCCATGTGTATCACTGGCAAACTGTGATGGACGACACCGTCAGTGCGTCCGTCGCGCAGGCTCTCGATGAGCTGATGCTTTGGGCCGAGGACTGCCCCGAAGTCCGGCACCTCGTGCACGCGGATTTCGGCTCCAACAATGTCCTGACGGACAATGGCCGCATAACAGCGGTCATTGACTGGAGCGAGGCGATGTTCGGGGATTCCCAATACGAGGTCGCCAACATCTTCTTCTGGAGGCCGTGGTTGGCTTGTATGGAGCAGCAGACGCGCTACTTCGAGCGGAGGCATCCGGAGCTTGCAGGATCGCCGCGGCTCCGGGCGTATATGCTCCGCATTGGTCTTGACCAACTCTATCAGAGCTTGGTTGACGGCAATTTCGATGATGCAGCTTGGGCGCAGGGTCGATGCGACGCAATCGTCCGATCCGGAGCCGGGACTGTCGGGCGTACACAAATCGCCCGCAGAAGCGCGGCCGTCTGGACCGATGGCTGTGTAGAAGTACTCGCCGATAGTGGAAACCGACGCCCCAGCACTCGTCCGAGGGCAAAGGAATAGCACGTACTACGAGATTTCGATTCCACCGCCGCCTTCTATGAAAGGTTGGGCTTCGGAATCGTTTTCCGGGACGCCGGCTGGATGATCCTCCAGCGCGGGGATCTCATGCTGGAGTTCTTCGCCCACCCCAACTTGTTTATTGCAGCTTATAATGGTTACAAATAAAGCAATAGCATCACAAATTTCACAAATAAAGCATTTTTTTCACTGCATTCTAGTTGTGGTTTGTCCAAACTCATCAATGTATCTTATCATGTCTGTATACCGTCGACCTCTAGCTAGAGCTTGGCGTAATCATGGTCATAGCTGTTTCCTGTGTGAAATTGTTATCCGCTCACAATTCCACACAACATACGAGCCGGAAGCATAAAGTGTAAAGCCTGGGGTGCCTAATGAGTGAGCTAACTCACATTAATTGCGTTGCGCTCACTGCCCGCTTTCCAGTCGGGAAACCTGTCGTGCCAGCTGCATTAATGAATCGGCCAACGCGCGGGGAGAGGCGGTTTGCGTATTGGGCGCTCTTCCGCTTCCTCGCTCACTGACTCGCTGCGCTCGGTCGTTCGGCTGCGGCGAGCGGTATCAGCTCACTCAAAGGCGGTAATACGGTTATCCACAGAATCAGGGGATAACGCAGGAAAGAACATGTGAGCAAAAGGCCAGCAAAAGGCCAGGAACCGTAAAAAGGCCGCGTTGCTGGCGTTTTTCCATAGGCTCCGCCCCCCTGACGAGCATCACAAAAATCGACGCTCAAGTCAGAGGTGGCGAAACCCGACAGGACTATAAAGATACCAGGCGTTTCCCCCTGGAAGCTCCCTCGTGCGCTCTCCTGTTCCGACCCTGCCGCTTACCGGATACCTGTCCGCCTTTCTCCCTTCGGGAAGCGTGGCGCTTTCTCATAGCTCACGCTGTAGGTATCTCAGTTCGGTGTAGGTCGTTCGCTCCAAGCTGGGCTGTGTGCACGAACCCCCCGTTCAGCCCGACCGCTGCGCCTTATCCGGTAACTATCGTCTTGAGTCCAACCCGGTAAGACACGACTTATCGCCACTGGCAGCAGCCACTGGTAACAGGATTAGCAGAGCGAGGTATGTAGGCGGTGCTACAGAGTTCTTGAAGTGGTGGCCTAACTACGGCTACACTAGAAGAACAGTATTTGGTATCTGCGCTCTGCTGAAGCCAGTTACCTTCGGAAAAAGAGTTGGTAGCTCTTGATCCGGCAAACAAACCACCGCTGGTAGCGGTGGTTTTTTTGTTTGCAAGCAGCAGATTACGCGCAGAAAAAAAGGATCTCAAGAAGATCCTTTGATCTTTTCTACGGGGTCTGACGCTCAGTGGAACGAAAACTCACGTTAAGGGATTTTGGTCATGAGATTATCAAAAAGGATCTTCACCTAGATCCTTTTAAATTAAAAATGAAGTTTTAAATCAATCTAAAGTATATATGAGTAAACTTGGTCTGACAGTTACCAATGCTTAATCAGTGAGGCACCTATCTCAGCGATCTGTCTATTTCGTTCATCCATAGTTGCCTGACTCCCCGTCGTGTAGATAACTACGATACGGGAGGGCTTACCATCTGGCCCCAGTGCTGCAATGATACCGCGAGACCCACGCTCACCGGCTCCAGATTTATCAGCAATAAACCAGCCAGCCGGAAGGGCCGAGCGCAGAAGTGGTCCTGCAACTTTATCCGCCTCCATCCAGTCTATTAATTGTTGCCGGGAAGCTAGAGTAAGTAGTTCGCCAGTTAATAGTTTGCGCAACGTTGTTGCCATTGCTACAGGCATCGTGGTGTCACGCTCGTCGTTTGGTATGGCTTCATTCAGCTCCGGTTCCCAACGATCAAGGCGAGTTACATGATCCCCCATGTTGTGCAAAAAAGCGGTTAGCTCCTTCGGTCCTCCGATCGTTGTCAGAAGTAAGTTGGCCGCAGTGTTATCACTCATGGTTATGGCAGCACTGCATAATTCTCTTACTGTCATGCCATCCGTAAGATGCTTTTCTGTGACTGGTGAGTACTCAACCAAGTCATTCTGAGAATAGTGTATGCGGCGACCGAGTTGCTCTTGCCCGGCGTCAATACGGGATAATACCGCGCCACATAGCAGAACTTTAAAAGTGCTCATCATTGGAAAACGTTCTTCGGGGCGAAAACTCTCAAGGATCTTACCGCTGTTGAGATCCAGTTCGATGTAACCCACTCGTGCACCCAACTGATCTTCAGCATCTTTTACTTTCACCAGCGTTTCTGGGTGAGCAAAAACAGGAAGGCAAAATGCCGCAAAAAAGGGAATAAGGGCGACACGGAAATGTTGAATACTCATACTCTTCCTTTTTCAATATTATTGAAGCATTTATCAGGGTTATTGTCTCATGAGCGGATACATATTTGAATGTATTTAGAAAAATAAACAAATAGGGGTTCCGCGCACATTTCCCCGAAAAGTGCCACCTGACGTC

> 4

GACGGATCGGGAGATCTCCCGATCCCCTATGGTGCACTCTCAGTACAATCTGCTCTGATGCCGCATAGTTAAGCCAGTATCTGCTCCCTGCTTGTGTGTTGGAGGTCGCTGAGTAGTGCGCGAGCAAAATTTAAGCTACAACAAGGCAAGGCTTGACCGACAATTGCATGAAGAATCTGCTTAGGGTTAGGCGTTTTGCGCTGCTTCGCGATGTACGGGCCAGATATACGCGTTGACATTGATTATTGACTAGTTATTAATAGTAATCAATTACGGGGTCATTAGTTCATAGCCCATATATGGAGTTCCGCGTTACATAACTTACGGTAAATGGCCCGCCTGGCTGACCGCCCAACGACCCCCGCCCATTGACGTCAATAATGACGTATGTTCCCATAGTAACGCCAATAGGGACTTTCCATTGACGTCAATGGGTGGAGTATTTACGGTAAACTGCCCACTTGGCAGTACATCAAGTGTATCATATGCCAAGTACGCCCCCTATTGACGTCAATGACGGTAAATGGCCCGCCTGGCATTATGCCCAGTACATGACCTTATGGGACTTTCCTACTTGGCAGTACATCTACGTATTAGTCATCGCTATTACCATGGTGATGCGGTTTTGGCAGTACATCAATGGGCGTGGATAGCGGTTTGACTCACGGGGATTTCCAAGTCTCCACCCCATTGACGTCAATGGGAGTTTGTTTTGGCACCAAAATCAACGGGACTTTCCAAAATGTCGTAACAACTCCGCCCCATTGACGCAAATGGGCGGTAGGCGTGTACGGTGGGAGGTCTATATAAGCAGAGCTCTCCCTATCAGTGATAGAGATCTCCCTATCAGTGATAGAGATCGTCGACGAGCTCGTTTAGTGAACCGTCAGATCGCCTGGAGACGCCATCCACGCTGTTTTGACCTCCATAGAAGACACCGGGACCGATCCAGCCTCCGGACTCTAGCGTTTAAACTTAAGCTTggtaccatggtgagcaagggcgaggagctgttcaccggggtggtgcccatcctggtcgagctggacggcgacgtaaacggccacaagttcagcgtgtccggcgagggcgagggcgatgccacctacggcaagctgaccctgaagttcatctgcaccaccggcaagctgcccgtgccctggcccaccctcgtgAccaccctgacctacggcgtgcagtgcttcagccgctaccccgaccacatgaagcagcacgacttcttcaagtccgccatgcccgaaggctacgtccaggagcgcaccatcttcttcAaggacgacggcaactacaagacccgcgccgaggtgaagttcgagggcgacaccctggtgaaccgcatcgagctgaagggcatcgacttcaaggaggacggcaacatcctggggcacaagctggagtacaactacaacagccacaacgtctatatcatggccgacaagcagaagaacggcatcaaggtgaacttcaagatccgccacaacatcgaggacggcagcgtgcagctcgccgaccactaccagcagaacacccccatcggcgacggccccgtgctgctgcccgacaaccactacctgagcacccagtccgccctgagcaaagaccccaacgagaagcgcgatcacatggtcctgctggagttcgtgaccgccgccgggatcactctcggcatggacgagctgtacaagGGGATGGCATCATATCCCTGTCATCAACACGCGAGTGCTTTTGACCAAGCAGCCCGGTCTCGAGGACATAATAACCGACGCACAGCTCTTCGCCCTCGACGGCAACAGGAGGCGACAGAGGTCCGACCGGAGCAAAAGATGCCTACGCTGCTTCGGGTATATATTGACGGCCCGCATGGTATGGGCAAGACGACAACTACGCAACTGCTGGTCGCGTTGGGTAGCCGAGATGACATCGTCTATGTCCCAGAGCCGATGACATACTGGCGGGTGCTCGGAGCAAGCGAGACTATAGCGAACATATACACAACTCAACATCGACTTGATCAAGGCGAAATATCCGCCGGCGATGCGGCGGTGGTGATGACGTCAGCGCAAATTACGATGGGTATGCCCTACGCCGTTACAGATGCGGTTCTTGCTCCTCACATTGGCGGCGAAGCGGGGAGTTCACATGCGCCACCGCCGGCTTTGACGCTTATTTTCGACCGACACCCCATTGCGGCGCTTCTGTGTTACCCGGCGGCAAGGTACCTCATGGGCTCTATGACGCCCCAAGCCGTACTGGCATTTGTCGCCCTTATTCCCCCTACTCTGCCCGGTACGAACATAGTACTTGGAGCGTTGCCTGAAGATAGGCACATTGATCGCCTGGCAAAGAGACAACGGCCCGGCGAACGACTCGATTTGGCTATGCTCGCTGCGATTAGGCGGGTGTACGGGCTGCTGGCGAACACGGTGCGATACCTCCAGTGCGGTGGTAGTTGGAGGGAGGATTGGGGTCAACTCAGCGGCACTGCAGTACCCCCCCAAGGCGCCGAGCCGCAGTCTAATGCCGGCCCACGACCCCACATCGGCGACACACTCTTTACCCTCTTTAGAGCTCCAGAACTGCTTGCTCCCAACGGTGACCTTTACAACGTCTTCGCGTGGGCACTGGACGTCCTGGCGAAGCGACTTCGCTCCATGCACGTGTTCATTCTTGATTACGACCAGTCACCAGCCGGATGCAGAGACGCCCTTTTGCAGTTGACCTCTGGAATGGTTCAGgttttttatgaggtagaaaggtcaacgtctgaatctcagtacagtgtacagagcagcactatgcttaagttttagcctttgttaaaagctttgctttagtacttttactcgagaaatgcagagtagaaagttaaatcagattctaaggttctgtgtcttaatcaatttaatgtgtaactggatttctgttcaaatattctagaatataaatgggatttaaatgataacttggaatattaataataaaatgtgttttatttttaaacattgaatgaggccaggcgcagtagctcacgcctgtaatcccaacacttcaggaggccaaggcaggcagatcacctgagatccagcctggccaacatggtgaaccccgtctctaccagaaataaaaatttagccggttgtggtggtgcacgcctgtagtcccagctactggcaggtgaattgcttgaacccggcaggcagaggttgcagtgaatttagattgcaccactgcactccactgtaggcaacagagcgagagactcaaaaaaaaaaataaataaatgaatacatagtgaaagcaaaattataggctgaggcaggggaattgcttgagcccgggaatttgagaccagcctgagcagcatagggaacctccacaaaaaatgaggcaggaagccggggaggttgaagctgcagtgagccaagtacgctcccacctgagtctcctcccccagagcaagaccccaccttaaaagaaaaaattatagcctctgtcttagccttttaaaagcaaatttataatggcttataaaagcaaatAAGATAACCGGTTACCAACTGCGGCCGCCtcgacttcataagccattttggaaagccaacacttttcaggcagtttcttgtaatctcttaatgcatgcatcgtacactgagggcaggtaaaatggatgccttgaaagagtccagagaataattttaaagatgttaatttagtatgacaagggtatgtggctttttatttttgccaaaaatttggtatttcggggtttgggtagggaaatgtcaatcatagtttaaaaataaagatatctttgagaatacttccaaagataaaaatgaagtaaaattgtgacattttggtcctcccttgtcaaaatgtgtaatttgacaagtcaaataatgtagttagtaagtattgaccacagttgtcattgataatggtattgaggtatatggatgtctattttgtgcattatttggcaatgtttaagaagttaacagttgtcagtcattgtctgaaagtgagtctgtaagtgtattgttcagcattttatcacccatgaaaaccctttttttatttaaagcatcactctattgaatggtactaaaggtacaaatttgttggggctgagatttttttcttagtttgatttttttttttaagagcatttacttaggaatgttctgtgttctaatactacttatttcagccaccagagagaaccctatgccagtttctaaaaagggtggcaaatcacttgacctgcaagaaacatctcattcatgctctatgaaagtttttattttactaaatgaatgacataattttttaaaacttacaaactgaatgcgtgttgtgttgcctttctgtcttcacagACTCACGTCACGACTCCAGGTTCTATACCAACGATCTGTGATCTCGCTCGAACATTCGCGAGAGAAATGGGTGAGGCCAACTGAGCTCGAGTCTAGAGGGCCCGTTTAAACCCGCTGATCAGCCTCGACTGTGCCTTCTAGTTGCCAGCCATCTGTTGTTTGCCCCTCCCCCGTGCCTTCCTTGACCCTGGAAGGTGCCACTCCCACTGTCCTTTCCTAATAAAATGAGGAAATTGCATCGCATTGTCTGAGTAGGTGTCATTCTATTCTGGGGGGTGGGGTGGGGCAGGACAGCAAGGGGGAGGATTGGGAAGACAATAGCAGGCATGCTGGGGATGCGGTGGGCTCTATGGCTTCTGAGGCGGAAAGAACCAGCTGGGGCTCTAGGGGGTATCCCCACGCGCCCTGTAGCGGCGCATTAAGCGCGGCGGGTGTGGTGGTTACGCGCAGCGTGACCGCTACACTTGCCAGCGCCCTAGCGCCCGCTCCTTTCGCTTTCTTCCCTTCCTTTCTCGCCACGTTCGCCGGCTTTCCCCGTCAAGCTCTAAATCGGGGGCTCCCTTTAGGGTTCCGATTTAGTGCTTTACGGCACCTCGACCCCAAAAAACTTGATTAGGGTGATGGTTCACGTACCTAGAAGTTCCTATTCCGAAGTTCCTATTCTCTAGAAAGTATAGGAACTTCCTTGGCCAAAAAGCCTGAACTCACCGCGACGTCTGTCGAGAAGTTTCTGATCGAAAAGTTCGACAGCGTCTCCGACCTGATGCAGCTCTCGGAGGGCGAAGAATCTCGTGCTTTCAGCTTCGATGTAGGAGGGCGTGGATATGTCCTGCGGGTAAATAGCTGCGCCGATGGTTTCTACAAAGATCGTTATGTTTATCGGCACTTTGCATCGGCCGCGCTCCCGATTCCGGAAGTGCTTGACATTGGGGAgTTCAGCGAGAGCCTGACCTATTGCATCTCCCGCCGTGCACAGGGTGTCACGTTGCAAGACCTGCCTGAAACCGAACTGCCCGCTGTTCTGCAGCCGGTCGCGGAGGCCATGGATGCGATCGCTGCGGCCGATCTTAGCCAGACGAGCGGGTTCGGCCCATTCGGACCGCAAGGAATCGGTCAATACACTACATGGCGTGATTTCATATGCGCGATTGCTGATCCCCATGTGTATCACTGGCAAACTGTGATGGACGACACCGTCAGTGCGTCCGTCGCGCAGGCTCTCGATGAGCTGATGCTTTGGGCCGAGGACTGCCCCGAAGTCCGGCACCTCGTGCACGCGGATTTCGGCTCCAACAATGTCCTGACGGACAATGGCCGCATAACAGCGGTCATTGACTGGAGCGAGGCGATGTTCGGGGATTCCCAATACGAGGTCGCCAACATCTTCTTCTGGAGGCCGTGGTTGGCTTGTATGGAGCAGCAGACGCGCTACTTCGAGCGGAGGCATCCGGAGCTTGCAGGATCGCCGCGGCTCCGGGCGTATATGCTCCGCATTGGTCTTGACCAACTCTATCAGAGCTTGGTTGACGGCAATTTCGATGATGCAGCTTGGGCGCAGGGTCGATGCGACGCAATCGTCCGATCCGGAGCCGGGACTGTCGGGCGTACACAAATCGCCCGCAGAAGCGCGGCCGTCTGGACCGATGGCTGTGTAGAAGTACTCGCCGATAGTGGAAACCGACGCCCCAGCACTCGTCCGAGGGCAAAGGAATAGCACGTACTACGAGATTTCGATTCCACCGCCGCCTTCTATGAAAGGTTGGGCTTCGGAATCGTTTTCCGGGACGCCGGCTGGATGATCCTCCAGCGCGGGGATCTCATGCTGGAGTTCTTCGCCCACCCCAACTTGTTTATTGCAGCTTATAATGGTTACAAATAAAGCAATAGCATCACAAATTTCACAAATAAAGCATTTTTTTCACTGCATTCTAGTTGTGGTTTGTCCAAACTCATCAATGTATCTTATCATGTCTGTATACCGTCGACCTCTAGCTAGAGCTTGGCGTAATCATGGTCATAGCTGTTTCCTGTGTGAAATTGTTATCCGCTCACAATTCCACACAACATACGAGCCGGAAGCATAAAGTGTAAAGCCTGGGGTGCCTAATGAGTGAGCTAACTCACATTAATTGCGTTGCGCTCACTGCCCGCTTTCCAGTCGGGAAACCTGTCGTGCCAGCTGCATTAATGAATCGGCCAACGCGCGGGGAGAGGCGGTTTGCGTATTGGGCGCTCTTCCGCTTCCTCGCTCACTGACTCGCTGCGCTCGGTCGTTCGGCTGCGGCGAGCGGTATCAGCTCACTCAAAGGCGGTAATACGGTTATCCACAGAATCAGGGGATAACGCAGGAAAGAACATGTGAGCAAAAGGCCAGCAAAAGGCCAGGAACCGTAAAAAGGCCGCGTTGCTGGCGTTTTTCCATAGGCTCCGCCCCCCTGACGAGCATCACAAAAATCGACGCTCAAGTCAGAGGTGGCGAAACCCGACAGGACTATAAAGATACCAGGCGTTTCCCCCTGGAAGCTCCCTCGTGCGCTCTCCTGTTCCGACCCTGCCGCTTACCGGATACCTGTCCGCCTTTCTCCCTTCGGGAAGCGTGGCGCTTTCTCATAGCTCACGCTGTAGGTATCTCAGTTCGGTGTAGGTCGTTCGCTCCAAGCTGGGCTGTGTGCACGAACCCCCCGTTCAGCCCGACCGCTGCGCCTTATCCGGTAACTATCGTCTTGAGTCCAACCCGGTAAGACACGACTTATCGCCACTGGCAGCAGCCACTGGTAACAGGATTAGCAGAGCGAGGTATGTAGGCGGTGCTACAGAGTTCTTGAAGTGGTGGCCTAACTACGGCTACACTAGAAGAACAGTATTTGGTATCTGCGCTCTGCTGAAGCCAGTTACCTTCGGAAAAAGAGTTGGTAGCTCTTGATCCGGCAAACAAACCACCGCTGGTAGCGGTGGTTTTTTTGTTTGCAAGCAGCAGATTACGCGCAGAAAAAAAGGATCTCAAGAAGATCCTTTGATCTTTTCTACGGGGTCTGACGCTCAGTGGAACGAAAACTCACGTTAAGGGATTTTGGTCATGAGATTATCAAAAAGGATCTTCACCTAGATCCTTTTAAATTAAAAATGAAGTTTTAAATCAATCTAAAGTATATATGAGTAAACTTGGTCTGACAGTTACCAATGCTTAATCAGTGAGGCACCTATCTCAGCGATCTGTCTATTTCGTTCATCCATAGTTGCCTGACTCCCCGTCGTGTAGATAACTACGATACGGGAGGGCTTACCATCTGGCCCCAGTGCTGCAATGATACCGCGAGACCCACGCTCACCGGCTCCAGATTTATCAGCAATAAACCAGCCAGCCGGAAGGGCCGAGCGCAGAAGTGGTCCTGCAACTTTATCCGCCTCCATCCAGTCTATTAATTGTTGCCGGGAAGCTAGAGTAAGTAGTTCGCCAGTTAATAGTTTGCGCAACGTTGTTGCCATTGCTACAGGCATCGTGGTGTCACGCTCGTCGTTTGGTATGGCTTCATTCAGCTCCGGTTCCCAACGATCAAGGCGAGTTACATGATCCCCCATGTTGTGCAAAAAAGCGGTTAGCTCCTTCGGTCCTCCGATCGTTGTCAGAAGTAAGTTGGCCGCAGTGTTATCACTCATGGTTATGGCAGCACTGCATAATTCTCTTACTGTCATGCCATCCGTAAGATGCTTTTCTGTGACTGGTGAGTACTCAACCAAGTCATTCTGAGAATAGTGTATGCGGCGACCGAGTTGCTCTTGCCCGGCGTCAATACGGGATAATACCGCGCCACATAGCAGAACTTTAAAAGTGCTCATCATTGGAAAACGTTCTTCGGGGCGAAAACTCTCAAGGATCTTACCGCTGTTGAGATCCAGTTCGATGTAACCCACTCGTGCACCCAACTGATCTTCAGCATCTTTTACTTTCACCAGCGTTTCTGGGTGAGCAAAAACAGGAAGGCAAAATGCCGCAAAAAAGGGAATAAGGGCGACACGGAAATGTTGAATACTCATACTCTTCCTTTTTCAATATTATTGAAGCATTTATCAGGGTTATTGTCTCATGAGCGGATACATATTTGAATGTATTTAGAAAAATAAACAAATAGGGGTTCCGCGCACATTTCCCCGAAAAGTGCCACCTGACGTC

> 5

GACGGATCGGGAGATCTCCCGATCCCCTATGGTGCACTCTCAGTACAATCTGCTCTGATGCCGCATAGTTAAGCCAGTATCTGCTCCCTGCTTGTGTGTTGGAGGTCGCTGAGTAGTGCGCGAGCAAAATTTAAGCTACAACAAGGCAAGGCTTGACCGACAATTGCATGAAGAATCTGCTTAGGGTTAGGCGTTTTGCGCTGCTTCGCGATGTACGGGCCAGATATACGCGTTGACATTGATTATTGACTAGTTATTAATAGTAATCAATTACGGGGTCATTAGTTCATAGCCCATATATGGAGTTCCGCGTTACATAACTTACGGTAAATGGCCCGCCTGGCTGACCGCCCAACGACCCCCGCCCATTGACGTCAATAATGACGTATGTTCCCATAGTAACGCCAATAGGGACTTTCCATTGACGTCAATGGGTGGAGTATTTACGGTAAACTGCCCACTTGGCAGTACATCAAGTGTATCATATGCCAAGTACGCCCCCTATTGACGTCAATGACGGTAAATGGCCCGCCTGGCATTATGCCCAGTACATGACCTTATGGGACTTTCCTACTTGGCAGTACATCTACGTATTAGTCATCGCTATTACCATGGTGATGCGGTTTTGGCAGTACATCAATGGGCGTGGATAGCGGTTTGACTCACGGGGATTTCCAAGTCTCCACCCCATTGACGTCAATGGGAGTTTGTTTTGGCACCAAAATCAACGGGACTTTCCAAAATGTCGTAACAACTCCGCCCCATTGACGCAAATGGGCGGTAGGCGTGTACGGTGGGAGGTCTATATAAGCAGAGCTCTCCCTATCAGTGATAGAGATCTCCCTATCAGTGATAGAGATCGTCGACGAGCTCGTTTAGTGAACCGTCAGATCGCCTGGAGACGCCATCCACGCTGTTTTGACCTCCATAGAAGACACCGGGACCGATCCAGCCTCCGGACTCTAGCGTTTAAACTTAAGCTTggtaccatggtgagcaagggcgaggagctgttcaccggggtggtgcccatcctggtcgagctggacggcgacgtaaacggccacaagttcagcgtgtccggcgagggcgagggcgatgccacctacggcaagctgaccctgaagttcatctgcaccaccggcaagctgcccgtgccctggcccaccctcgtgAccaccctgacctacggcgtgcagtgcttcagccgctaccccgaccacatgaagcagcacgacttcttcaagtccgccatgcccgaaggctacgtccaggagcgcaccatcttcttcAaggacgacggcaactacaagacccgcgccgaggtgaagttcgagggcgacaccctggtgaaccgcatcgagctgaagggcatcgacttcaaggaggacggcaacatcctggggcacaagctggagtacaactacaacagccacaacgtctatatcatggccgacaagcagaagaacggcatcaaggtgaacttcaagatccgccacaacatcgaggacggcagcgtgcagctcgccgaccactaccagcagaacacccccatcggcgacggccccgtgctgctgcccgacaaccactacctgagcacccagtccgccctgagcaaagaccccaacgagaagcgcgatcacatggtcctgctggagttcgtgaccgccgccgggatcactctcggcatggacgagctgtacaagGGGATGGCATCATATCCCTGTCATCAACACGCGAGTGCTTTTGACCAAGCAGCCCGGTCTCGAGGACATAATAACCGACGCACAGCTCTTCGCCCTCGACGGCAACAGGAGGCGACAGAGGTCCGACCGGAGCAAAAGATGCCTACGCTGCTTCGGGTATATATTGACGGCCCGCATGGTATGGGCAAGACGACAACTACGCAACTGCTGGTCGCGTTGGGTAGCCGAGATGACATCGTCTATGTCCCAGAGCCGATGACATACTGGCGGGTGCTCGGAGCAAGCGAGACTATAGCGAACATATACACAACTCAACATCGACTTGATCAAGGCGAAATATCCGCCGGCGATGCGGCGGTGGTGATGACGTCAGCGCAAATTACGATGGGTATGCCCTACGCCGTTACAGATGCGGTTCTTGCTCCTCACATTGGCGGCGAAGCGGGGAGTTCACATGCGCCACCGCCGGCTTTGACGCTTATTTTCGACCGACACCCCATTGCGGCGCTTCTGTGTTACCCGGCGGCAAGGTACCTCATGGGCTCTATGACGCCCCAAGCCGTACTGGCATTTGTCGCCCTTATTCCCCCTACTCTGCCCGGTACGAACATAGTACTTGGAGCGTTGCCTGAAGATAGGCACATTGATCGCCTGGCAAAGAGACAACGGCCCGGCGAACGACTCGATTTGGCTATGCTCGCTGCGATTAGGCGGGTGTACGGGCTGCTGGCGAACACGGTGCGATACCTCCAGTGCGGTGGTAGTTGGAGGGAGGATTGGGGTCAACTCAGCGGCACTGCAGTACCCCCCCAAGGCGCCGAGCCGCAGTCTAATGCCGGCCCACGACCCCACATCGGCGACACACTCTTTACCCTCTTTAGAGCTCCAGAACTGCTTGCTCCCAACGGTGACCTTTACAACGTCTTCGCGTGGGCACTGGACGTCCTGGCGAAGCGACTTCGCTCCATGCACGTGTTCATTCTTGATTACGACCAGTCACCAGCCGGATGCAGAGACGCCCTTTTGCAGTTGACCTCTGGAATGGTTCAGgtaagttatgaggtagaaaggtcaacgtctgaatctcagtacagtgtacagagcagcactatgcttaagttttagcctttgttaaaagctttgctttagtacttttactcgagaaatgcagagtagaaagttaaatcagattctaaggttctgtgtcttaatcaatttaatgtgtaactggatttctgttcaaatattctagaatataaatgggatttaaatgataacttggaatattaataataaaatgtgttttatttttaaacattgaatgaggccaggcgcagtagctcacgcctgtaatcccaacacttcaggaggccaaggcaggcagatcacctgagatccagcctggccaacatggtgaaccccgtctctaccagaaataaaaatttagccggttgtggtggtgcacgcctgtagtcccagctactggcaggtgaattgcttgaacccggcaggcagaggttgcagtgaatttagattgcaccactgcactccactgtaggcaacagagcgagagactcaaaaaaaaaaataaataaatgaatacatagtgaaagcaaaattataggctgaggcaggggaattgcttgagcccgggaatttgagaccagcctgagcagcatagggaacctccacaaaaaatgaggcaggaagccggggaggttgaagctgcagtgagccaagtacgctcccacctgagtctcctcccccagagcaagaccccaccttaaaagaaaaaattatagcctctgtcttagccttttaaaagcaaatttataatggcttataaaagcaaatAAGATAACCGGTTACCAACTGCGGCCGCCtcgacttcataagccattttggaaagccaacacttttcaggcagtttcttgtaatctcttaatgACGTcatcgtacactgagggcaggtaaaatggatgccttgaaagagtccagagaataattttaaagatgttaatttagtatgacaagggtatgtggctttttatttttgccaaaaatttggtatttcggggtttgggtagggaaatgtcaatcatagtttaaaaataaagatatctttgagaatacttccaaagataaaaatgaagtaaaattgtgacattttggtcctcccttgtcaaaatgtgtaatttgacaagtcaaataatgtagttagtaagtattgaccacagttgtcattgataatggtattgaggtatatggatgtctattttgtgcattatttggcaatgtttaagaagttaacagttgtcagtcattgtctgaaagtgagtctgtaagtgtattgttcagcattttatcacccatgaaaaccctttttttatttaaagcatcactctattgaatggtactaaaggtacaaatttgttggggctgagatttttttcttagtttgatttttttttttaagagcatttacttaggaatgttctgtgttctaatactacttatttcagccaccagagagaaccctatgccagtttctaaaaagggtggcaaatcacttgacctgcaagaaacatctcattcatgctctatgaaagtttttattttactaaatgaatgacataattttttaaaacttacaaactgaatgcgtgttgtgttgcctttctgtcttcacagACTCACGTCACGACTCCAGGTTCTATACCAACGATCTGTGATCTCGCTCGAACATTCGCGAGAGAAATGGGTGAGGCCAACTGAGCTCGAGTCTAGAGGGCCCGTTTAAACCCGCTGATCAGCCTCGACTGTGCCTTCTAGTTGCCAGCCATCTGTTGTTTGCCCCTCCCCCGTGCCTTCCTTGACCCTGGAAGGTGCCACTCCCACTGTCCTTTCCTAATAAAATGAGGAAATTGCATCGCATTGTCTGAGTAGGTGTCATTCTATTCTGGGGGGTGGGGTGGGGCAGGACAGCAAGGGGGAGGATTGGGAAGACAATAGCAGGCATGCTGGGGATGCGGTGGGCTCTATGGCTTCTGAGGCGGAAAGAACCAGCTGGGGCTCTAGGGGGTATCCCCACGCGCCCTGTAGCGGCGCATTAAGCGCGGCGGGTGTGGTGGTTACGCGCAGCGTGACCGCTACACTTGCCAGCGCCCTAGCGCCCGCTCCTTTCGCTTTCTTCCCTTCCTTTCTCGCCACGTTCGCCGGCTTTCCCCGTCAAGCTCTAAATCGGGGGCTCCCTTTAGGGTTCCGATTTAGTGCTTTACGGCACCTCGACCCCAAAAAACTTGATTAGGGTGATGGTTCACGTACCTAGAAGTTCCTATTCCGAAGTTCCTATTCTCTAGAAAGTATAGGAACTTCCTTGGCCAAAAAGCCTGAACTCACCGCGACGTCTGTCGAGAAGTTTCTGATCGAAAAGTTCGACAGCGTCTCCGACCTGATGCAGCTCTCGGAGGGCGAAGAATCTCGTGCTTTCAGCTTCGATGTAGGAGGGCGTGGATATGTCCTGCGGGTAAATAGCTGCGCCGATGGTTTCTACAAAGATCGTTATGTTTATCGGCACTTTGCATCGGCCGCGCTCCCGATTCCGGAAGTGCTTGACATTGGGGAgTTCAGCGAGAGCCTGACCTATTGCATCTCCCGCCGTGCACAGGGTGTCACGTTGCAAGACCTGCCTGAAACCGAACTGCCCGCTGTTCTGCAGCCGGTCGCGGAGGCCATGGATGCGATCGCTGCGGCCGATCTTAGCCAGACGAGCGGGTTCGGCCCATTCGGACCGCAAGGAATCGGTCAATACACTACATGGCGTGATTTCATATGCGCGATTGCTGATCCCCATGTGTATCACTGGCAAACTGTGATGGACGACACCGTCAGTGCGTCCGTCGCGCAGGCTCTCGATGAGCTGATGCTTTGGGCCGAGGACTGCCCCGAAGTCCGGCACCTCGTGCACGCGGATTTCGGCTCCAACAATGTCCTGACGGACAATGGCCGCATAACAGCGGTCATTGACTGGAGCGAGGCGATGTTCGGGGATTCCCAATACGAGGTCGCCAACATCTTCTTCTGGAGGCCGTGGTTGGCTTGTATGGAGCAGCAGACGCGCTACTTCGAGCGGAGGCATCCGGAGCTTGCAGGATCGCCGCGGCTCCGGGCGTATATGCTCCGCATTGGTCTTGACCAACTCTATCAGAGCTTGGTTGACGGCAATTTCGATGATGCAGCTTGGGCGCAGGGTCGATGCGACGCAATCGTCCGATCCGGAGCCGGGACTGTCGGGCGTACACAAATCGCCCGCAGAAGCGCGGCCGTCTGGACCGATGGCTGTGTAGAAGTACTCGCCGATAGTGGAAACCGACGCCCCAGCACTCGTCCGAGGGCAAAGGAATAGCACGTACTACGAGATTTCGATTCCACCGCCGCCTTCTATGAAAGGTTGGGCTTCGGAATCGTTTTCCGGGACGCCGGCTGGATGATCCTCCAGCGCGGGGATCTCATGCTGGAGTTCTTCGCCCACCCCAACTTGTTTATTGCAGCTTATAATGGTTACAAATAAAGCAATAGCATCACAAATTTCACAAATAAAGCATTTTTTTCACTGCATTCTAGTTGTGGTTTGTCCAAACTCATCAATGTATCTTATCATGTCTGTATACCGTCGACCTCTAGCTAGAGCTTGGCGTAATCATGGTCATAGCTGTTTCCTGTGTGAAATTGTTATCCGCTCACAATTCCACACAACATACGAGCCGGAAGCATAAAGTGTAAAGCCTGGGGTGCCTAATGAGTGAGCTAACTCACATTAATTGCGTTGCGCTCACTGCCCGCTTTCCAGTCGGGAAACCTGTCGTGCCAGCTGCATTAATGAATCGGCCAACGCGCGGGGAGAGGCGGTTTGCGTATTGGGCGCTCTTCCGCTTCCTCGCTCACTGACTCGCTGCGCTCGGTCGTTCGGCTGCGGCGAGCGGTATCAGCTCACTCAAAGGCGGTAATACGGTTATCCACAGAATCAGGGGATAACGCAGGAAAGAACATGTGAGCAAAAGGCCAGCAAAAGGCCAGGAACCGTAAAAAGGCCGCGTTGCTGGCGTTTTTCCATAGGCTCCGCCCCCCTGACGAGCATCACAAAAATCGACGCTCAAGTCAGAGGTGGCGAAACCCGACAGGACTATAAAGATACCAGGCGTTTCCCCCTGGAAGCTCCCTCGTGCGCTCTCCTGTTCCGACCCTGCCGCTTACCGGATACCTGTCCGCCTTTCTCCCTTCGGGAAGCGTGGCGCTTTCTCATAGCTCACGCTGTAGGTATCTCAGTTCGGTGTAGGTCGTTCGCTCCAAGCTGGGCTGTGTGCACGAACCCCCCGTTCAGCCCGACCGCTGCGCCTTATCCGGTAACTATCGTCTTGAGTCCAACCCGGTAAGACACGACTTATCGCCACTGGCAGCAGCCACTGGTAACAGGATTAGCAGAGCGAGGTATGTAGGCGGTGCTACAGAGTTCTTGAAGTGGTGGCCTAACTACGGCTACACTAGAAGAACAGTATTTGGTATCTGCGCTCTGCTGAAGCCAGTTACCTTCGGAAAAAGAGTTGGTAGCTCTTGATCCGGCAAACAAACCACCGCTGGTAGCGGTGGTTTTTTTGTTTGCAAGCAGCAGATTACGCGCAGAAAAAAAGGATCTCAAGAAGATCCTTTGATCTTTTCTACGGGGTCTGACGCTCAGTGGAACGAAAACTCACGTTAAGGGATTTTGGTCATGAGATTATCAAAAAGGATCTTCACCTAGATCCTTTTAAATTAAAAATGAAGTTTTAAATCAATCTAAAGTATATATGAGTAAACTTGGTCTGACAGTTACCAATGCTTAATCAGTGAGGCACCTATCTCAGCGATCTGTCTATTTCGTTCATCCATAGTTGCCTGACTCCCCGTCGTGTAGATAACTACGATACGGGAGGGCTTACCATCTGGCCCCAGTGCTGCAATGATACCGCGAGACCCACGCTCACCGGCTCCAGATTTATCAGCAATAAACCAGCCAGCCGGAAGGGCCGAGCGCAGAAGTGGTCCTGCAACTTTATCCGCCTCCATCCAGTCTATTAATTGTTGCCGGGAAGCTAGAGTAAGTAGTTCGCCAGTTAATAGTTTGCGCAACGTTGTTGCCATTGCTACAGGCATCGTGGTGTCACGCTCGTCGTTTGGTATGGCTTCATTCAGCTCCGGTTCCCAACGATCAAGGCGAGTTACATGATCCCCCATGTTGTGCAAAAAAGCGGTTAGCTCCTTCGGTCCTCCGATCGTTGTCAGAAGTAAGTTGGCCGCAGTGTTATCACTCATGGTTATGGCAGCACTGCATAATTCTCTTACTGTCATGCCATCCGTAAGATGCTTTTCTGTGACTGGTGAGTACTCAACCAAGTCATTCTGAGAATAGTGTATGCGGCGACCGAGTTGCTCTTGCCCGGCGTCAATACGGGATAATACCGCGCCACATAGCAGAACTTTAAAAGTGCTCATCATTGGAAAACGTTCTTCGGGGCGAAAACTCTCAAGGATCTTACCGCTGTTGAGATCCAGTTCGATGTAACCCACTCGTGCACCCAACTGATCTTCAGCATCTTTTACTTTCACCAGCGTTTCTGGGTGAGCAAAAACAGGAAGGCAAAATGCCGCAAAAAAGGGAATAAGGGCGACACGGAAATGTTGAATACTCATACTCTTCCTTTTTCAATATTATTGAAGCATTTATCAGGGTTATTGTCTCATGAGCGGATACATATTTGAATGTATTTAGAAAAATAAACAAATAGGGGTTCCGCGCACATTTCCCCGAAAAGTGCCACCTGACGTC
